# Cerebrovascular Function in Women With Polycystic Ovary Syndrome: A Pilot Multi‐Parameter Magnetic Resonance Imaging Study

**DOI:** 10.1111/cen.70067

**Published:** 2025-12-01

**Authors:** Melissa E. Wright, Cory T. Richards, Saajan Davies, Rachel N. Lord, D. Aled Rees, Kevin Murphy

**Affiliations:** ^1^ Cardiff University Brain Research Imaging Centre (CUBRIC), School of Physics and Astronomy Cardiff University Cardiff UK; ^2^ School of Sport and Health Sciences Cardiff Metropolitan University Cardiff UK; ^3^ School of Education, Health and Science University of Gloucestershire Cheltenham UK; ^4^ Neuroscience and Mental Health Innovation Institute Cardiff University Cardiff UK

**Keywords:** cerebrovascular function, insulin resistance, neuroimaging, PCOS, testosterone

## Abstract

**Objective:**

Polycystic ovary syndrome (PCOS) is associated with an increased risk of cerebrovascular disease, but the effects on cerebrovascular function are unknown. In this pilot study, we sought to compare cerebrovascular perfusion, pulsatility, reactivity and metabolism between women with PCOS and healthy volunteers using MRI, and investigated the influence of testosterone and insulin resistance on these parameters.

**Design:**

Case‐control pilot study.

**Patients:**

Fifteen patients with PCOS (age: 32.0 ± 7.4 years; body mass index [BMI]: 31.8 ± 5.7 kg/m^2^) and 12 healthy controls (HC) (age: 30.7 ± 6.4 years; BMI: 30.2 ± 5.8 kg/m^2^).

**Measurements:**

We used 3T magnetic resonance imaging (MRI) to assess several aspects of cerebrovascular function: (1) perfusion (cerebral blood flow [CBF] and arterial arrival time [AAT]; PCOS *N* = 15; HC *N* = 12), (2) pulsatility index (PCOS *N* = 15; HC *N* = 12), (3) breath‐hold induced cerebrovascular reactivity (CVR; PCOS *N* = 15; HC N = 10), and (4) global oxygen metabolism (oxygen extraction fraction [OEF] and cerebral metabolic rate of oxygen [CMRO_2_]; PCOS *N* = 9; HC *N* = 8). Linear regression models investigated the contribution of PCOS status, serum testosterone and Homeostatic Model Assessment for Insulin Resistance (HOMA2‐IR). Regional analysis underwent false discovery rate (FDR) correction for multiple comparisons.

**Results:**

Overall baseline CBF was not statistically different in PCOS patients compared to controls after adjustment for other variables (χ^2^(1) = 3.29; *p* = 0.07) but did show evidence for regional reduction with PCOS status in the transverse temporal gyrus (χ^2^(84) = 110.31; *p* = 0.03; −29.05 mL/100 g/min ± 7.26 [standard error; SE]). Similarly, while PCOS status was not associated with overall CVR (χ^2^(1) = 0.78; *p* = 0.38), there was evidence of a regional interaction (χ^2^(84) = 154.25; *p* < 0.001) in the parahippocampus (1.37% signal change ± 0.27 [SE]) and pericalcarine cortex (1.04% signal change ± 0.26 [SE]). Neither testosterone nor HOMA2‐IR was associated with any outcome measure.

**Conclusions:**

We observed regional reduction in cerebral blood flow and regional increase in cerebrovascular reactivity in women with PCOS compared to healthy controls. However, due to the limited statistical power and unclear menstrual timing in this pilot study, these results require further replication.

## 1 Introduction

Polycystic ovary syndrome (PCOS) is a common, multi‐system condition affecting 10%–13% of the premenopausal population [1]. In addition to its reproductive manifestations, PCOS is recognised as a metabolic disorder with long‐term health risks, including an increased risk of cerebrovascular disease [2]. This may contribute to reduced cognitive ability in early [3, 4] and mid‐life [5], as well as an increased risk of major adverse cardiovascular events and stroke [6]. Potential mediators may include direct effects of hyperandrogenism [7] or hyperinsulinaemia [8] on the vascular system.

Previous studies of cerebrovascular structure and function in individuals with PCOS have shown mixed results, with greater carotid intima‐media thickness, lower carotid pulsatility and reduced vascular reactivity in patients with PCOS reported in some [9, 10, 11], but not all [12] studies. Cerebral metabolism may also be altered: in an ^18^F‐FDG PET imaging study of lean patients with PCOS, the cerebral metabolic rate of glucose in frontal, parietal, and temporal cortices was 9%–14% lower than controls [13]. White matter lesions, often associated with small vessel disease [14], have also been shown to be more prevalent among women with PCOS [15]. Whilst these studies hint at altered cerebrovascular physiology in women with PCOS, no studies have examined this comprehensively, nor considered the potential underlying mechanisms, such as hyperandrogenism. For example, in the periphery, arterial stiffness as measured via the cardio‐ankle vascular index (CAVI), was found to be higher in women with PCOS compared to controls, which was independently associated with excess androgens [7]. Insulin resistance is also a biochemical hallmark of most women with PCOS and might also be implicated in altered cerebrovascular function. In keeping with this contention, cerebral perfusion is lower in adults with metabolic syndrome compared to controls [16, 17], a reduction that is more apparent when comparing women with and without metabolic syndrome than their male counterparts [16].

We hypothesised that global and regional cerebrovascular flow and function would be reduced in women with PCOS. To investigate this, and generate more specific, region‐dependent hypotheses, this pilot study aimed to capture a range of cerebrovascular parameters using advanced neuroimaging and tested associations with possible contributing factors (i.e., testosterone and insulin resistance). Modern advances in MRI facilitate interrogation of cerebrovascular function in unprecedented detail, offering major advantages on traditional measures such as Doppler ultrasound.

## 2 Materials and Methods

### 2.1 Participants

Fifteen women with PCOS and 13 age‐ and body mass index (BMI)‐matched controls were recruited. Approval was given by the NHS Research Ethics Committee (REC:19/WA/0093) and institutional ethical boards (Cardiff Metropolitan University: STA‐9540; Cardiff University: EC.19.12.10.5916A). The study was registered on ClinicalTrials.gov (NCT05394935) and followed the Declaration of Helsinki. Patients with PCOS were recruited through the endocrinology clinic at the University Hospital of Wales, Cardiff, via social media and the PCOS charity, Verity. Healthy volunteers were recruited via word of mouth and social media. All participants provided written informed consent. Healthy volunteers self‐reported a regular menstrual cycle (between 27 and 31 days). All testing sessions occurred during the early follicular phase of their menstrual cycles, based on the self‐reported first day of menses (day 1–7). No such temporal limitations were applied in oligomenorrhoeic or amenorrhoeic women with PCOS. Since hormonal contraceptives alter the endocrine profile, hormonal contraceptive use was recorded for modelling.

Participants fasted for a minimum of 4 h and were required to abstain from caffeine, alcohol and strenuous physical activity for 24 h before the neuroimaging session to avoid any potential influence on haemodynamic parameters.

### 2.2 Inclusion/Exclusion Criteria

A diagnosis of PCOS was made in accordance with the Rotterdam criteria, including exclusion of congenital adrenal hyperplasia, Cushing's syndrome, thyroid disease, hyperprolactinaemia and androgen‐secreting tumours through clinical and/or biochemical evaluation. All participants did not meet recommended physical activity guidelines of 150 min of moderate, or 75 min of vigorous physical activity per week [18]. Participants were excluded if they had any of the commonly accepted contraindications to MRI scanning (e.g., ferromagnetic devices), a clinically significant condition (such as other metabolic, neurological, cardiovascular, psychiatric, cerebrovascular, or respiratory condition), were pregnant (or had been in the last 6 weeks), were breastfeeding, or were post‐menopausal. Control participants were additionally excluded if they had clinical or biochemical evidence of hyperandrogenism. One control participant was excluded on the basis of biochemical hyperandrogenism, leaving a final control sample of 12. Participants taking medication for PCOS, insulin‐sensitising drugs, oral contraceptives (OCP) or anti‐anxiety/antidepression medications were required to be on a stable dose for 3 months before study entry.

### 2.3 Anthropometric and Biochemical Measurements

Anthropometric measurements were undertaken at Cardiff Metropolitan University. Participant height and weight were measured using a fixed stadiometer (Holtain Ltd., Pembrokeshire, U.K.) and scales (SECA 770, Vogel & Halke, Hamburg, Germany) respectively. Body Mass Index (BMI) was calculated as weight (kg)/height (m)^2^. Blood sampling and analysis was completed at the University Hospital Wales. Serum total testosterone and androstenedione concentrations were measured by liquid chromatography‐tandem mass spectrometry (Quattro Premier XE triple quadrupole mass spectrometer), and sex hormone binding globulin (SHBG) using an Alinity i reagent kit (Abbott Diagnostics, Illinois, USA). Fasting glucose, total cholesterol, HDL‐cholesterol, LDL‐cholesterol and triglycerides were measured using an Alinity c reagent kit (Abbott Diagnostics, Illinois, USA), while fasting insulin was measured using a Mercodia Iso‐Insulin ELISA kit (Mercodia, Sweden). Free androgen index (FAI) was calculated as testosterone (nmol/l)/SHBG (nmol/l) x100. The Homeostatic Model Assessment for Insulin Resistance (HOMA2‐IR) was generated using ‘HOMA2 Calculator V.2.2.3.’ software (http://www.dtu.ox.ac.uk/homacalculator/index.php [19]).

### 2.4 MRI Acquisition

Full scan parameters are available in the Supporting Information S1. Briefly, the following metrics were acquired at Cardiff University Brain Research Imaging Centre (CUBRIC) on a Siemens MAGNETOM Prisma 3T scanner:

**Structure:** a high‐resolution structural T1 image using an MPRAGE (Magnetization‐Prepared Rapid Acquisition Gradient Echo) sequence (1 mm^3^; repetition time [TR] = 2.1 s; echo time [TE] = 3.24 ms), from which individual labels of cortical and subcortical areas were created (regions‐of‐interest [ROIs]).

**Cerebral blood flow (CBF) and arterial arrival time (AAT):** measured using a multi post‐labelling delay pseudocontinuous arterial spin labelling (MPLD‐pCASL) scan (maximum TR = 5.6 s; TE = 11 s; voxel resolution = 3.4 × 3.4 × 6.0 mm). CBF is defined as the volume of blood (in millilitres) reaching 100 g of brain tissue per minute delivering sufficient oxygen/nutrients for neural function. AAT is the duration it takes for blood to arrive at brain tissue (from a labelling plane in the carotid) and is informative of any blood supply delays.

**Pulsatility Index (PI):** measured in the internal carotid arteries using dynamic inflow magnitude contrast (DIMAC) [20], with a 15 ms temporal resolution. PI reflects blood flow resistance in the cerebral arteries, calculated using the difference between peak systolic and end‐diastolic velocities, and is related to changes in vessel stiffness.

**Breath‐hold cerebrovascular reactivity (CVR):** a blood oxygen level dependent (BOLD) echo planar imaging (EPI) scan (TR = 1 s; TE = 30 ms; voxel size = 2.0 mm^3^) was completed during a 9‐min breath‐hold task (detailed below). CVR is the brain's ability to adjust blood flow in response to a vasodilating stimulus (here, breath‐hold induced fluctuations in CO_2_ were utilised) and is a key indicator of cerebrovascular reserve capacity and health.

**Global oxygen extraction fraction (OEF) and cerebral metabolic rate of oxygen (CMRO**_**2**_ **):** oxygen metabolism metrics were collected in a subsample (PCOS *n* = 9, control *n* = 8) using a T2‐relaxation‐under‐spin‐tagging (TRUST) [21] sequence (TR = 3 s; TE = 3.9 ms). A T1 inversion recovery sequence (ΔTR/TE = 150 ms/22 ms, flip angle = 90 degrees, and GRAPPA acceleration factor = 2; with 960 acquisitions > 16 repeats of 60 measurements) was also collected to quantify the T1 of venous blood, used to calculate haematocrit, which is used to estimate OEF and CMRO_2_. OEF represents the proportion of blood oxygen removed by brain tissue and is related to CMRO_2_, the quantified rate of oxygen metabolism. These metrics give information of global energy usage and metabolism, a critical aspect of overall cerebrovascular health. To generate a global value, this scan was localised over the sagittal sinus.

### 2.5 MRI Breath‐Hold Task

During the breath‐hold task, continuous partial pressure of end‐tidal oxygen (PETO_2_) and carbon dioxide (PETCO_2_) signals in expired air were measured via a nasal cannula (AEI Technologies, PA, US). Each breath‐hold‐task began with 20 s of rest. The following sequence was repeated 10 times with instructions appearing in the middle of the stimulus screen.

– 24 s of paced breathing (3 s in and 3 s out), ending on an ‘out’ prompt.
– 20 s of breath‐hold.
– 2 s of expelling air after completing the breath‐hold. This allows for the rise in CO_2_ to be measured.
– 4 s of recovery (spontaneous breathing with no specific instructions).

After all breath‐hold challenges were completed, another 24 s of resting data were recorded.

### 2.6 MRI Pre‐Processing

#### 2.6.1 Structure

A grey matter mask was generated for use in other processing pipelines. The T1‐weighted MPRAGE structural images were processed using the ‘fsl_anat’ pipeline [22], which includes reorientating to standard orientation, bias‐field correction, brain extraction, and tissue‐type segmentation.

#### 2.6.2 Cerebral Blood Flow (CBF) and Arterial Arrival Time (AAT)

Pre‐processing and generation of the perfusion maps were completed using the Analysis of Functional NeuroImages (AFNI) software package [23]. Following motion correction, the scan was split into separate post labelling delays (PLDs; five pairs of each) and the deltaM was calculated (tag minus control). For quantification, the M0 of the blood was calculated as:

$$M_{0}\mathrm{blood}=\frac{M_{0}\mathrm{csf}\;*\text{exp}(\mathrm{TE}*\left(\left(\frac{1}{T_{2}\mathrm{csf}}\right)-\left(\frac{1}{T_{2}\mathrm{blood}}\right)\right))}{\lambda \mathrm{csf}}$$

λcsf = blood‐CSF partition coefficient (1.15) [24, 25].
$M_{0}\mathrm{csf}$ = the M_0_ of the cerebral spinal fluid (CSF) was taken from a CSF mask generated around a manually positioned central point in the lateral ventricles.
TE = echo time (0.019 s).
$T_{2}\mathrm{csf}$ = T_2_* of CSF (0.4 s) [24].
$T_{2}\mathrm{blood}$ = T_2_* of blood (0.06 s) [26].

The 3dNLfim command and the Buxton model [27] was used for final quantification. CBF values were allowed to vary between 0 and 300, and AAT values could vary between 0 and 3. The final CBF and AAT maps were thresholded by R^2^ > 0.6 (excluding a sample average 93% of voxels, which includes voxels outside of the brain).

FLIRT [22] was used to generate a transformation matrix to register the Desikan‐Killiany Atlas (cortical and subcortical regions) to each participant's native space [28]. The cortical regions were further restricted to the individual's grey matter voxels, using their individual grey matter mask. The median CBF and AAT were extracted for each region. The median CBF and AAT across all atlas voxels were taken as global measures for comparison.

#### 2.6.3 Pulsatility Index (PI)

The DIMAC time series for left and right internal carotid artery were generated by averaging the signal from a 4 × 4 voxel square region over each artery. A 3 s cut‐off high‐pass filter and 5th‐order (21 timepoint frame length) Savitzky‐Golay low‐pass filter was applied. Each pulse period was then modelled using Fourier analysis, with five sine/cosine pairs with periods ranging from one to one fifth of the pulse period. PI was calculated as the range divided by the mean of the modelled timeseries for each pulse period, then averaged across pulse periods.

#### 2.6.4 Breath‐Hold Cerebrovascular Reactivity (CVR)

Similar to the pCASL perfusion maps, pre‐processing (e.g., motion correction, brain extraction, slice time correction, conversion to % change) of the Blood Oxygen Level Dependent (BOLD) signal timeseries was completed using the AFNI software package [23]. PETCO_2_ traces were processed using in‐house scripts. This involves clipping the traces to only include the relevant scan, rescaling to mmHg, and converting the trace to a haemodynamic‐response‐function [HRF]‐convolved regressor. The final regressor is cross‐correlated (Spearman's Rho) to the global BOLD time course (with 41 incremental time shifts in CO_2_ trace) to find the best temporal match between time series, defined as the correlation with the highest value. This allows for the breath‐hold induced increases in PETCO_2_ and BOLD to be matched and the reactivity to be modelled. The final PETCO_2_ trace was used to model the CVR response using AFNI. A threshold of *R* ^2^ > 0.06 was applied to the final CVR maps to exclude noisy voxels (excluding a sample average 89% of voxels, which includes voxels outside of the brain). The same process to extract global and ROI median values used for the perfusion maps was applied.

Due to a technical error in PETCO_2_ recording, two controls were excluded from the CVR analysis at this point, leaving 10 healthy controls for CVR analysis.

#### 2.6.5 Global Oxygen Extraction Fraction (OEF) and Cerebral Metabolic Rate of Oxygen (CMRO_2_)

Both metrics were estimated using TRUST scan data. The T_2_ of blood was calculated by nonlinear least‐squared fitting of a mono‐exponential curve, as a function of the effective echo times. The two most intense voxels were used and venous oxygenation (Yv) was estimated by inverting the relationship between Yv, haemoglobin and T_2_ [21]. The OEF value was then used with grey matter CBF for the CMRO_2_ calculation (see Supporting Information S1). SaO_2_ was assumed to be 98%, as has been used previously [29].

### 2.7 Statistical Analysis

Participant characteristics were compared using independent *t*‐tests, computed with JASP software [30]. A student's *t*‐test was used, unless a significant Shapiro‐Wilk test indicated non‐normality in one or both groups, in which case a Mann‐Whitney *t*‐test was employed.

Linear models were constructed using R software (v.4.2.2) [31] and the ‘lmerTest’ analysis package [32]. Statistical significance was set at *p* < 0.05. Three tiers of linear models with additional fixed effects were used to investigate contributions of PCOS‐related factors to cerebrovascular outcomes:

*Status model*. Firstly, PCOS status was added alone to maximise statistical power and investigate any overall group differences.

*Status/hormonal contraceptive model*. Hormonal contraceptive use was then added to account for possible impact on endocrine function.

*Status/hormonal contraceptive/testosterone/HOMA2‐IR model*. Testosterone variance and insulin resistance (HOMA2‐IR) were then also added, to investigate how these hallmarks of PCOS contribute to cerebrovascular outcome variance.

In all models, participant was included as a random effect and, if relevant, ROI/laterality was added as an additional fixed crossed effect. Residual plots were visually inspected and showed no notable deviations from homoscedasticity or normality. To test if an outcome contributed to a model, an ANOVA was used to compare the model with and without that outcome variable. If relevant, an interaction with cortical ROI was then added to the model; considering the limited statistical power and hypothesis‐generating aim of this pilot study, all regions were considered exploratory. If a statistically significant interaction was found, the ROI individual coefficients were examined and a false discovery rate (FDR) correction for multiple comparisons was employed using the Benjamini‐Hochberg (BH) procedure [33] and correcting over all 83 regional comparisons. All ROI comparison data, including *p*‐values and adjusted q‐values, are provided in the Supporting Information S1.

For CVR, CBF, and AAT, all ROIs that had 0 voxels that passed the R^2^ threshold were excluded from model fitting (109/2100 observations for CVR, 13/2268 observations for perfusion maps).

Power calculations were generated for a wider study on peripheral blood flow changes in PCOS and based on inducing a significant increase of MCAv during exercise (Δ15.5 cm·s^−1^) [34]. These a priori power estimates suggested a sample of 14 per group with an α = 0.05 and β = 0.80.

### 2.8 Sensitivity Analysis

Sensitivity analysis was carried out for medicated compared to non‐medicated individuals. As there was such a small, homogeneous medicated sample, these were combined for this analysis (hormonal contraceptives, metformin, psychotropics, anti‐androgens). For each vascular outcome, linear models were constructed in which medication status was a primary fixed effect, participant was a random effect and, if relevant, ROI/laterality was added as an additional fixed effect. We then examined whether medication status explained a significant amount of outcome variance in each model.

For all quality group differences tests, either a Student's independent *t*‐test or Mann‐Whitney *t*‐test was used, depending on normality checks, via JASP v.0.17 software [30]. Due to the potential influence of task‐related effort and ventilatory drive, breath hold CVR‐related quality metrics were extracted and group differences investigated. These metrics were:

– ΔPETCO_2_—defined as the max‐min end tidal CO_2_ recording across the breath hold scan.
– CO_2_‐BOLD Rho—the Rho statistic from the Spearman's Rho correlation between the optimum PETCO_2_ trace and the BOLD time course, indicating the direction and strength of the relationship.
– Optimal CO_2_‐BOLD lag—the time lag that the PETCO_2_ trace was shifted to get the optimal correlation with the BOLD time course (defined as the highest Rho value).

An additional fixed effect of per‐subject ΔPETCO_2_ was added to any statistically significant CVR model to investigate if any group differences were driven by breath‐hold amplitude.

TRUST quality check metrics (goodness‐of‐fit adjusted R^2^ value and venous oxygenation [Yv]) were also extracted and group differences compared similarly.

Finally, quality check metrics for DIMAC‐derived PI was investigated. Firstly, the within‐session coefficient of variation (CV) was calculated across the whole sample (defined as standard deviation/mean) to describe overall variation. Next, test‐retest reliability was investigated. The DIMAC timeseries for every participant was divided into two (1st compared to 2nd half) and analysed separately, including the vessel selection and PI calculation. Intraclass correlations (ICC) were used to calculate test‐retest reliability (left and right vessels averaged).

## 3 Results

### 3.1 Clinical and Biochemical Characteristics

The demographic and clinical characteristics for the full study population are shown in Table 1. The PCOS and control groups were well‐matched for age and BMI (nonsignificant difference found in the full sample, CVR sample, and TRUST subsample; all *p* > 0.05). As anticipated, the PCOS group had higher levels of circulating testosterone, free androgen index and androstenedione compared to controls (all *p* < 0.05). However, HOMA2‐IR was not different between groups. The rate of hormonal contraceptive use was higher in the control group compared to the PCOS group (PCOS = 0; controls = 3; out of these, two were on a hormonal contraceptive pill and one a contraceptive injection).

**Table 1:** Clinical characteristics and biochemical data.

	PCOS	Controls	Test statistic	*p* value
N	15	12		
Age (years)	32.0 ± 7.4	30.7 ± 6.4	−0.45	0.654
Body Mass Index (kg/m^2^)	31.8 ± 5.7	30.2 ± 5.8	−0.73	0.475
Hormonal contraceptive (N,%)	0, 0%	3, 25%		
Metaformin use (N,%)	0, 0%	1, 8.3%		
Psychotropic use (N,%)	3, 20%	3, 25%		
Anti‐androgen use (N,%)	0, 0%	1, 8.3%		
Androstenedione (nmol/L)	5.3 ± 1.8	3.8 ± 1.5	−*2.20*	*0.037*
Sex Hormone Binding Globulin (nmol/L)	41.5 ± 15.7	56.3 ± 40.8	1.30	0.206
Testosterone (nmol/L)	1.4 ± 0.5	1.1 ± 0.3	−*2.09*	*0.047*
Free Androgen Index (%)	3.6 ± 1.4	2.4 ± 1.3	−*2.25*	*0.034*
Cholesterol (mmol/L)	5.0 ± 0.9	4.6 ± 1.0	−0.90	0.377
Triglycerides (mmol/L)	1.0 ± 0.4	0.9 ± 0.3	−1.14	0.264
HDL cholesterol (mmol/L)	1.4 ± 0.2	1.5 ± 0.3	1.00	0.327
Non‐HDL cholesterol (mmol/L)	3.6 ± 0.9	3.2 ± 0.8	−1.28	0.212
LDL cholesterol (mmol/L)	3.1 ± 0.8	2.8 ± 0.7	−1.14	0.264
Fasting glucose (mmol/L)	4.7 ± 0.5	4.7 ± 0.4	0.16	0.875
Fasting Insulin (μU/mL)	9.9 ± 6.2	9.2 ± 3.3	91.50*	0.961
HOMA2‐IR	1.3 ± 0.8	1.2 ± 0.4	92.50*	0.922

*Note:* Data are reported as mean ± SD. All statistics are independent Student *t*‐tests, unless indicated by a *, in which case the data were non‐normally distributed and a Mann‐Whitney U test was used. Significant group differences (at the *p* < 0.05 level) are shown in italics.

Abbreviations: HDL, High‐density lipoprotein; HOMA2‐IR, Homeostatic Model of Insulin Resistance; LDL, Low‐density lipoprotein.

### 3.2 Outlier Detection

**Figure 1:**
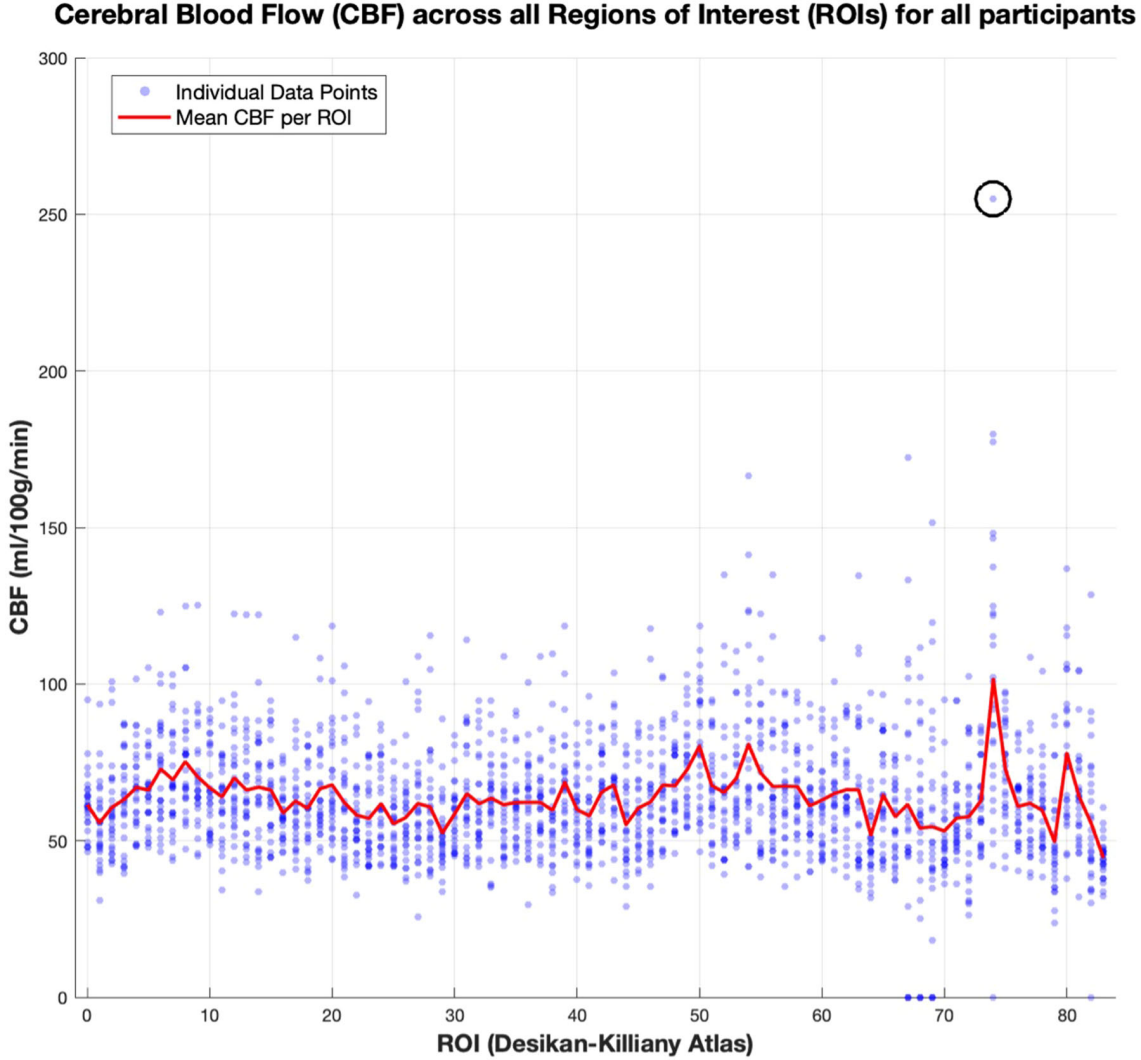
Plot of cerebral blood flow (CBF) values against region of interest (ROI) labels. Each single datapoint is a CBF value from a single ROI from an individual (all ROIs and individuals included). Desikan‐Killiany atlas ID labels used for ROIs, with 0 = global. The red line shows the mean average across ROIs. The outlier in ROI 74 (left transverse temporal gyrus) is shown in the unfilled circle.

Post‐hoc visual inspection of the CBF data revealed an outlier (defined as three standard deviations from the mean for that ROI; see Figure 1) that was high enough to be biologically implausible. It was therefore excluded and analysis repeated (for the initial analysis with outlier included, see Supporting Information S1). We also examined whether the results were robust to outlier exclusion. As a sensitivity analysis, CBF models were calculated with (a) the full sample, (b) the sample excluding the outlier value, and (c) the full sample with a robust mixed‐effect model (Huber weighting) that will reduce the impact of the outlier. The pattern of the results (i.e., directionality of the estimates and standard errors) remained consistent, suggesting a degree of robustness to the results (full results in Supporting Information S1).

### 3.3 MRI Outcomes

**Figure 2:**
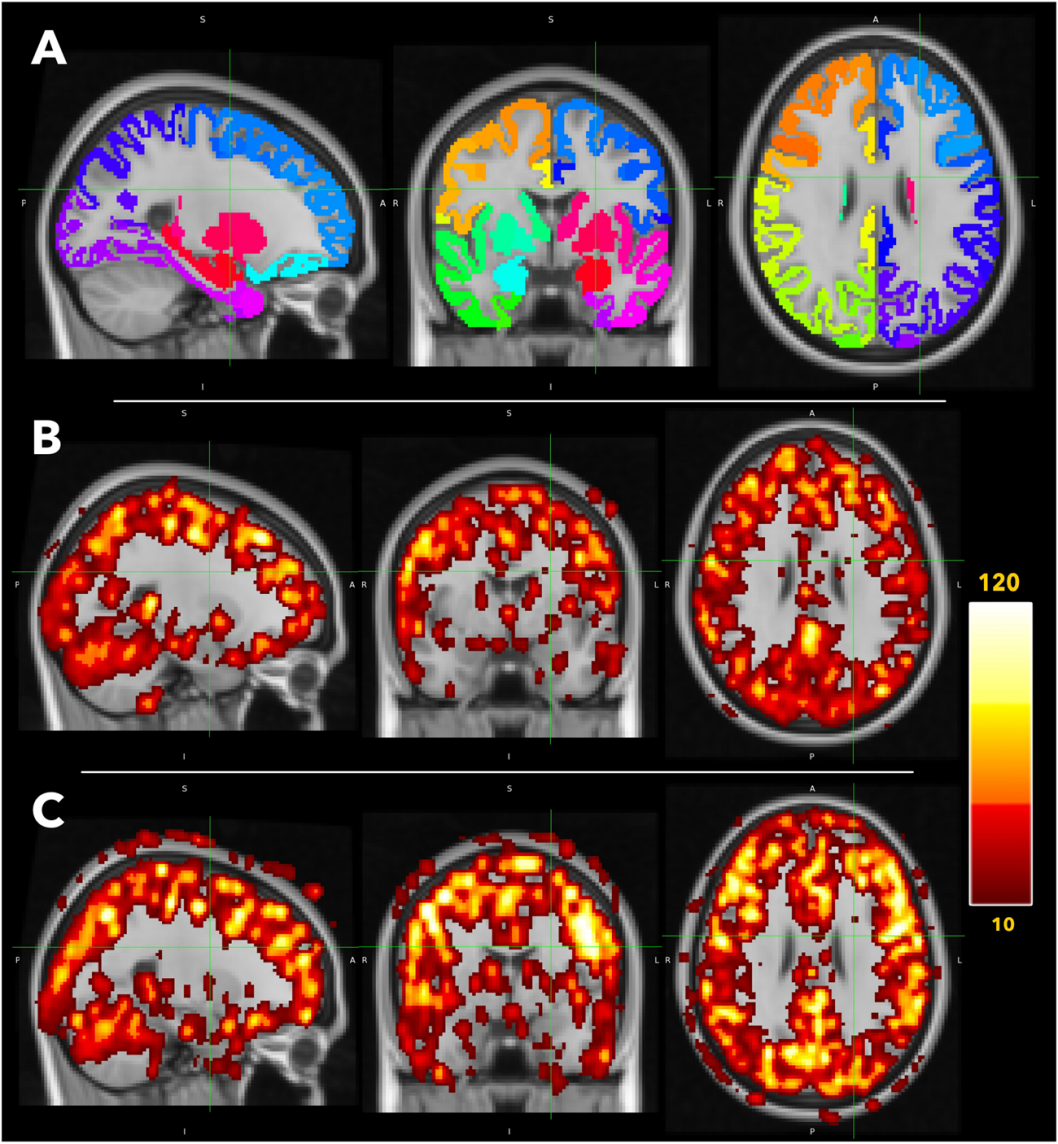
Example MRI data. (A) illustrates the Desikan‐Killiany Atlas (Desikan et al. 2006) used to mask and delineate perfusion and reactivity data. Different colours indicate different cortical or subcortical regions of interest. (B and C) show the cerebral blood flow (CBF) data for a representative PCOS patient and healthy control respectively. Data is smoothed by a 2 mm gaussian kernel for visualisation purposes only. Darker colours indicate lower CBF and lighter colours indicate higher CBF. The scale is in ml/100 g/min. The data are converted to MNI (Montreal Neurological Institute) standard space for visualisation.

**Figure 3:**
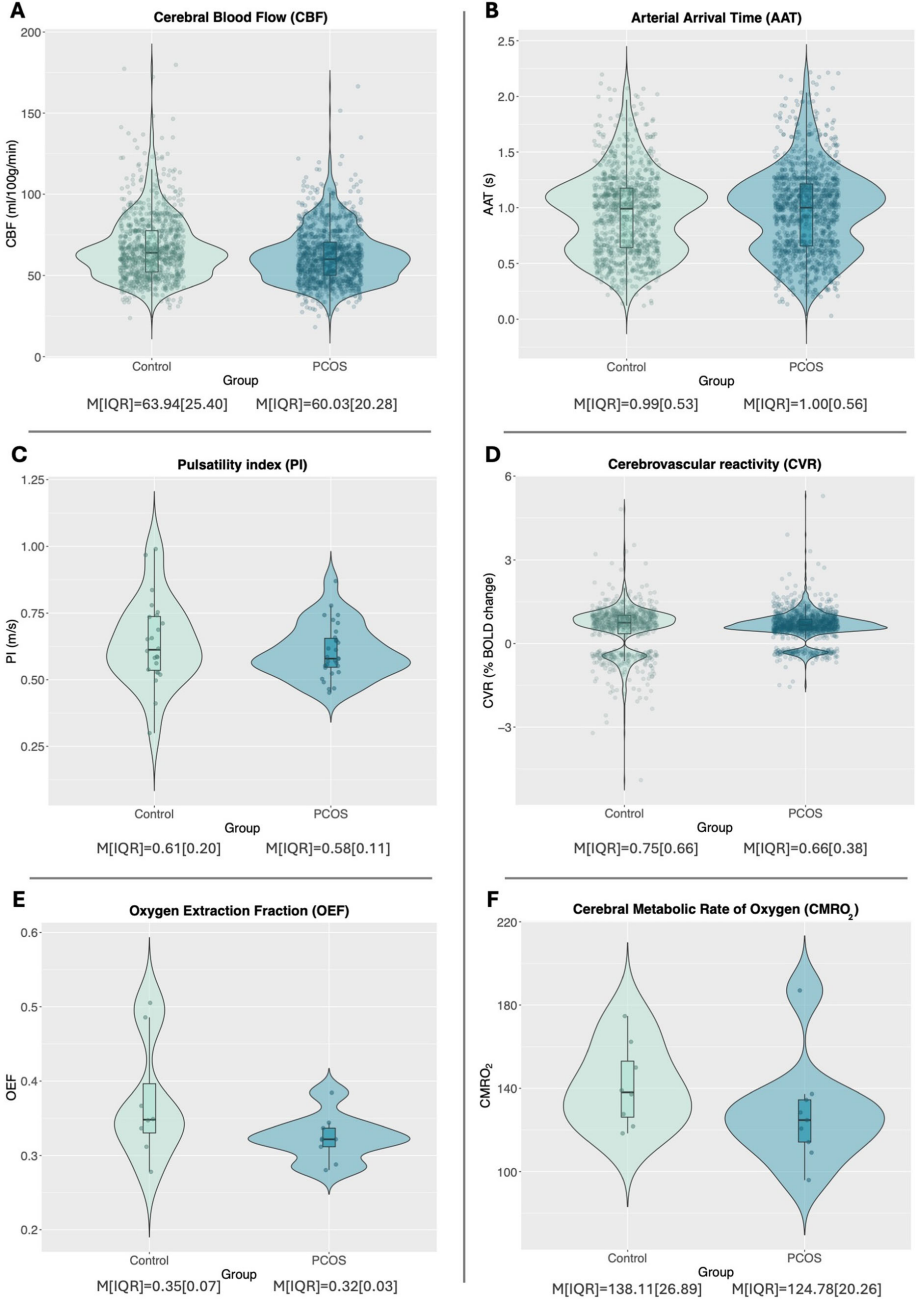
Violin plots comparing (A) cerebral blood flow (CBF; mL/100 g/min), (B) arterial arrival time (AAT; seconds), (C) pulsatility index (PI; m/s), (D) cerebrovascular reactivity (CVR; % BOLD change with each mmHg increase in CO_2_) values from all regions of interest (ROIs) between the control group (left, green) and the PCOS group (right, blue). The final line are violin plots comparing (E) global cerebral metabolic rate of oxygen (CMRO_2_) and (F) global oxygen extraction fraction (OEF) between the healthy control group (left, green), and the PCOS group (right, blue). The box‐and‐whisker plots indicate the median (central line), first and third quartile (hinges), and 1.5 *interquartile range from the hinge (whiskers). Individual datapoints are shown as filled circles. Median (M) and inter‐quartile range (IQR) are presented. Data presented in A–C are from 15 PCOS patients and 12 healthy controls, data presented in D are from 15 PCOS patients and 10 healthy controls, and data in E and F are from 9 PCOS patients and 8 healthy controls.

An example of the CBF data for a PCOS and control participant is shown in Figure 2. Summary results are plotted by group in Figure 3.

Results are presented in Tables 2, 3, 4. Residual plots were visually inspected and the assumptions of homoscedasticity or normality held in all models. We found that PCOS status did not significantly contribute to overall global CBF variance, which persisted in models with and without the other explanatory variables (hormonal contraceptive, testosterone, and insulin resistance). However, there was evidence of a regional effect (*χ* ^*2*^ = 110.31; df = 1; *p* = 0.029); participants with PCOS had a significantly reduced CBF value in the left transverse temporal gyrus compared to healthy controls (t(2227.04) = −3.99; *p* = 0.005; estimate[standard error, SE] = −29.05[7.26]; 95% confidence intervals [CIs]= −43.29, −14.81). This was reflected in the CVR results: while no model suggested an overall influence of PCOS on global CVR, there was evidence of a regional effect (*χ* ^*2*^ = 154.25; df = 1; *p*‐value < 0.001). PCOS patients demonstrated a small increase in CVR compared to healthy controls in the left parahippocampus (t(1966.20) = 5.10; *p* < 0.001; estimate [SE] = 1.37 [0.27]; 95% CIs = 0.84, 1.90) and left pericalcarine cortex (t(1966.08) = 4.06; *p* = 0.002; estimate[SE] = 1.04 [0.26]; 95% CIs = 0.54, 1.54). There was also evidence of a regional influence of hormonal contraceptive use on brain CVR values (*χ* ^*2*^ = 337.49; df = 1; *p*‐value < 0.001), but none passed correction for multiple comparisons, and this should be interpreted with caution considering the small sample of participants on a form of hormonal contraceptives and the mix of hormonal formulations included. Neither serum testosterone nor HOMA2‐IR explained a significant amount of CBF or CVR variance. No other statistically significant effect was found in either the pulsatility, arterial arrival time, or the preliminary oxygen metabolism metrics.

**Table 2:** Model results for main MRI cerebrovascular outcomes.

		Model 1			Model 2			Model 3		
	Outcome	*χ* ^*2*^	*p* value	Estimate (SE)	*χ* ^*2*^	*p* value	Estimate (SE)	*χ* ^*2*^	*p* value	Estimate (SE)
CBF	PCOS status	1.48	0.224	−4.78 (3.88)	3.29	0.070	−7.50 (4.01)	3.29	0.070	−8.07 (4.31)
	Hormonal contraceptive use				2.79	0.095	−10.87 (6.34)	2.85	0.092	−11.03 (6.37)
	Testosterone							0.10	0.752	1.50 (4.77)
	HOMA2‐IR							0.01	0.936	0.25 (3.12)
AAT	PCOS status	0.58	0.447	0.01 (0.02)	0.21	0.644	0.010 (0.02)	0.70	0.404	0.02 (0.02)
	Hormonal contraceptive use				0.38	0.536	−0.02 (0.03)	0.22	0.640	−0.01 (0.03)
	Testosterone							0.37	0.546	−0.01 (0.02)
	HOMA2‐IR							1.85	0.396	−0.02 (0.02)
PI	PCOS status	0.68	0.411	−0.04 (0.04)	0.05	0.820	−0.01 (0.04)	0.07	0.797	−0.01 (0.05)
	Hormonal contraceptive use				1.94	0.164	0.10 (0.07)	2.08	0.150	0.10 (0.07)
	Testosterone							0.08	0.772	0.02 (0.05)
	HOMA2‐IR							2.23	0.328	−0.02 (0.07)
CVR	PCOS status	0.74	0.389	0.18 (0.21)	2.33	0.127	−0.23 (0.15)	0.85	0.356	−0.15 (0.16)
	Hormonal contraceptive use				**23.15**	**< 0.001**	**−1.38 (0.22)**	**23.56**	**< 0.001**	**−1.36 (0.22)**
	Testosterone							1.71	0.192	−0.22 (0.17)
	HOMA2‐IR							0.20	0.656	0.05 (0.10)

*Note:* Degrees‐of‐freedom (Df) = 1. Statistically significant results indicated in bold. CBF, AAT, and PI data are from 15 PCOS patients and 12 healthy controls, while CVR data are from 15 PCOS patients and 10 healthy controls.

Abbreviations: AAT, arterial arrival time; CBF, cerebral blood flow; CVR, cerebrovascular reactivity; PI, pulsatility index; SE, Standard Error.

**Table 3:** Model results for MRI oxygen metabolism outcomes.

		Model 1			Model 2			Model 3		
	Outcomes	t‐statistic	*p* value	Estimate (SE)	t‐statistic	*p* value	Estimate (SE)	t‐statistic	*p* value	Estimate (SE)
OEF	PCOS status	−1.70	0.110	−0.05 (0.03)	−1.27	0.226	−0.04 (0.03)	−1.24	0.130	−0.06 (0.04)
	Hormonal contraceptive use				0.53	0.493	0.03 (0.05)	0.628	0.628	0.03 (0.05)
	Testosterone							1.27	0.228	0.05 (0.04)
	HOMA2‐IR							−0.71	0.486	−0.02 (0.03)
CMRO_2_	PCOS status	−1.19	0.251	−13.40 (11.22)	−1.22	0.244	−15.23 (12.53)	−1.06	0.309	−16.42 (15.47)
	Hormonal contraceptive use				−0.38	0.711	−7.33 (19.41)	−0.37	0.720	−7.75 (21.11)
	Testosterone							0.13	0.901	2.04 (15.98)
	HOMA2−IR							0.05	0.962	0.52 (10.63)

*Note:* Model 1 degrees‐of‐freedom (Df) = 1,15. Model 2 Df = 2,14. Model 3 Df = 3,13. Data presented are from nine PCOS patients and eight healthy controls.

Abbreviations: CMRO_2_, cerebral metabolic rate of oxygen; OEF, oxygen extraction fraction; SE, Standard Error.

**Table 4:** Model results for brain regional effects of main MRI vascular outcomes (region*outcome interaction).

		Model 3 – brain regional effects					
	*Outcomes*	*χ* ^*2*^	*p* value	Region	t−statistic (Df)	Estimate (SE)	*p* value [corrected]
CBF	PCOS status	**110.31**	**0.029**	**Left transverse temporal gyrus**	**−3.99 (2227.04)**	**−29.05 (7.26)**	**0.005**
	Hormonal contraceptive use	46.68	1.00				
	Testosterone	76.99	0.693				
	HOMA2−IR	94.08	0.212				
AAT	PCOS status	98.93	0.127				
	Hormonal contraceptive use	76.04	0.720				
	Testosterone	71.53	0.832				
	HOMA2−IR	82.02	0.541				
CVR	PCOS status	**154.25**	**< 0.001**	**Left parahippocampus**	**5.10 (1966.20)**	**1.37 (0.27)**	**< 0.001**
				**Left pericalcarine cortex**	**4.06 (1966.08)**	**1.04 (0.26)**	**0.002**
	Hormonal contraceptive use	**155.38**	**< 0.001**				
	Testosterone	72.70	0.783				
	HOMA2−IR	38.67	> 0.99				

*Note:* Degrees‐of‐freedom (Df) = 84 unless otherwise stated. Statistically significant results indicated in bold. Regional *p*‐values corrected for multiple comparisons. CBF and AAT data are from 15 PCOS patients and 12 healthy controls, and CVR data are from 15 PCOS patients and 10 healthy controls.

Abbreviations: AAT, arterial arrival time; CBF, cerebral blood flow; CVR, cerebrovascular reactivity.

### 3.4 Sensitivity Analysis

Medication status alone did not significantly explain variance in any outcome variable in this sample (for full results, see Supporting Information S1).

Group differences in CVR‐related quality metrics were then investigated. Neither CO_2_‐BOLD Rho (t(23) = −1.23; *p* = 0.23) nor optimal CO_2_‐BOLD lag (W(23) = 73.50; *p* = 0.95) were statistically significantly different between groups. However, ΔPETCO_2_ was greater in PCOS patients compared to controls (t(23) = −2.23; *p* = 0.04; PCOS mean (SD) = 12.56 (3.46) mmHg; control mean (SD) = 9.45 (3.32) mmHg; figure in Supporting Information S1). The CVR analysis used accounts for such differences in ΔPETCO_2_ as individual PETCO_2_ traces were included in CVR modelling. Nevertheless, the final ROI‐interaction CVR model was repeated with per‐subject ΔPETCO_2_ as an additional fixed effect; if the regional group difference was no longer seen, it would suggest they were driven by breath‐hold amplitude variance. However, the pattern of results was maintained; there was evidence of a regional effect (*χ* ^*2*^ = 154.20; df = 1; *p*‐value < 0.001), with PCOS patients demonstrating a small increase in CVR compared to healthy controls in the left parahippocampus (t(1966) = 5.10; *p* < 0.001; estimate [SE] = 1.37[0.27]; 95% CIs = 0.84, 1.90) and left pericalcarine cortex (t(1966) = 4.05; *p* = 0.002; estimate [SE] = 1.03 [0.26]; 95% CIs = 0.53, 1.54).

TRUST goodness‐of fit values were > 0.99 for all participants. Neither Yv (t(15) = −1.70; *p* = 0.11) nor goodness‐of‐fit (W(15) = 55.00; *p* = 0.07) significantly differed between groups.

Finally, reliability of the PI dataset was investigated. A CV of 20% was found in the full dataset. A test‐retest ICC of 0.82 [95% CI: 0.65, 0.91) was calculated, which suggests good intrasession reliability.

## 4 Discussion

This preliminary study is the first to investigate the independent contribution of PCOS status, serum testosterone, and insulin resistance on a comprehensive range of cerebrovascular measures in a sample of PCOS patients and age‐and‐BMI‐matched controls, using advanced MRI. This pilot investigation aimed to broadly interrogate cerebrovascular flow and function so specific hypotheses could be generated for testing in subsequent adequately powered studies. We observed that PCOS diagnosis is associated with regional dampened cortical perfusion and regional enhanced cerebrovascular reactivity. Our observations have potentially important implications for our understanding of cerebrovascular risk in women with PCOS.

When considering the overall models and in line with recently published work using ultrasound (Richards et al. [35], Clinical Endocrinology – accepted), we found that PCOS did not significantly contribute to cerebrovascular function variance, with or without the addition of possible explanatory variables such as circulating testosterone or HOMA2‐IR. However, an additional in‐depth regional analysis suggested that women with PCOS had region‐specific reductions in CBF compared to healthy controls. This effect was significant despite accounting for testosterone and insulin resistance in the model, suggesting that additional mechanisms may drive this effect, such as wider endocrine or metabolic disruption. For example, patients with PCOS typically have relative progesterone deficiency, especially in the context of oligo‐/amenorrhoea. Since progesterone has been shown to increase CBF [36], relative progesterone deficiency may contribute to our observations. Additionally, women with PCOS demonstrated increases in regional CVR compared to controls. The difference between regional resting (CBF) and dynamic (CVR) function is notable and may suggest some region‐limited compensatory action, vascular dysregulation, or altered neurovascular coupling. In recent work from our lab (Richards et al. [35], Clinical Endocrinology – accepted), global neurovascular coupling measured using ultrasound was comparable between PCOS and healthy controls, but it may be important to interrogate regional variation to help understand PCOS cerebrovascular function. Since this was an exploratory study, these regions should be considered for future hypothesis‐driven work.

AAT and PI were also of interest as both may reflect vascular stiffness, an important marker of vascular ageing, and peripheral endothelial dysfunction has been observed in women with PCOS [37]. However, we found no effect of PCOS status on resting PI or AAT in this sample. While previous work has found an influence of serum testosterone concentration on vascular compliance, the evidence is much more inconclusive in women compared to men [38], suggesting that there may be sexually dimorphic effects. Previous work has found conflicting results when comparing PI in patients with PCOS to controls in a similar age range [11, 12]; however, the use of high‐resolution MRI techniques allows more accurate estimation. It is also possible that, in this age group, the effect of testosterone and insulin resistance on cerebrovascular stiffness is too subtle to be detected. This may not be the case in an older cohort with longer disease exposure than the cohort studied here, or if tested while the system was under stress, such as exercise. The effect of testosterone may also be dampened in our sample due to the relatively high BMI, as it has been previously suggested that the relationship between androgens and endothelial function reduces with weight gain [39].

Finally, we examined metrics of oxygen metabolism in a subsample. We found no influence of PCOS status on either OEF or CMRO_2_. This suggests that cortical oxygen metabolism is maintained in young adults with PCOS, although we recognise that we may have been underpowered to address this, and larger studies are needed.

This study formed part of a wider investigation of vascular health in PCOS in a ‘real world’ setting (Richards et al., 2025, Clinical Endocrinology – accepted). Medication used commonly in clinical practice was therefore included as long as participants had been taking them at a stable dose for at least 3 months. Though a sensitivity analysis suggested no statistically significant differences between individuals taking medication compared to those not taking medication, this may introduce noise to our vascular outcomes. Future research should therefore investigate outcomes in medication‐naïve cohorts. There are also other potential confounding factors that were not monitored and modelled but which could influence perfusion and reactivity outcomes, such as smoking status [40], blood pressure [41], dyslipidaemia [42], and obstructive sleep apnoea [43]. These factors should be included in future modelling work using a larger, more heterogeneous sample.

Our study has several strengths and some limitations. This is the first study to employ state‐of‐the‐art MRI techniques to interrogate a range of cerebrovascular measures in PCOS and examine associations with potential mechanisms. Age and BMI were carefully matched between groups, as obesity can independently impact cerebrovascular function [44]. Recruitment was also confined to sedentary individuals to reduce any impact of significant variation in physical fitness on vascular function. However, we acknowledge the relatively small sample size, especially with respect to measures of oxygen metabolism, which will reduce power for examining subtle region‐specific effects. These results should therefore be interpreted with caution and used to generate more targeted hypotheses. Additionally, insulin resistance did not differ between the PCOS and control groups, suggesting that the recruited PCOS sample had a relatively ‘mild’ metabolic phenotype. This may potentially explain why insulin resistance showed no relationship with our vascular measures, despite previous work demonstrating that insulin resistance may be linked to reduced cerebrovascular reactivity independently of BMI [45]. Our results may not thus be generalisable to patients with PCOS with a more severe metabolic phenotype. Additionally, the narrow age and BMI range means that our findings may not translate to older women or those with lean PCOS. However, this helped to specifically investigate PCOS‐associated effects. Finally, an important limitation is that, due to the presence of oligomenorrhoea or amenorrhoea in the PCOS patients, information on menstrual timing was not available and so the groups could not be matched in this aspect. This introduces additional noise in our PCOS vascular measurements that may contribute or mask group differences, as previous work has suggested that menstrual‐related fluctuations in oestrogen and progesterone are associated with small, global changes in cerebral blood flow [36].

In conclusion, our study demonstrates for the first time that PCOS patients have dampened regional resting CBF and elevated regional CVR compared to healthy controls, which were not associated with serum testosterone concentration or insulin resistance in this sample. However, due to the limited power of this pilot study, these results require further replication. Further studies with greater statistical power are needed to confirm our findings, investigate additional underlying mechanisms and establish whether measures which reduce vascular risk can improve cerebrovascular physiology in this population, especially as early cardiovascular risk factors are associated with cognitive impairment in later life [46].

## Supporting information


**Supporting Figure 1:** Violin plot comparing cerebral blood flow (CBF; ml/100g/min) values from all regions of interest (ROIs) between the healthy control group (left, green) and the PCOS group (right, blue). **Supporting Figure 2:** Violin plot comparing PETCO2 (defined as the max‐min end‐tidal CO2 trace recorded during the breath hold task) between the control group (left, green) and the PCOS group (right, blue). **Supporting Table 1:** Sensitivity analysis for outlier handling. **Supporting Table 2:** Regional interactions with PCOS status on cerebral blood flow. Q values are corrected for a false discovery rate (FDR) over 83 regions. **Supporting Table 3:** Regional interactions with PCOS status on cerebrovascular reactivity. Q values are corrected for a false discovery rate (FDR) over 83 regions. **Supporting Table 4:** Regional interactions with Hormonal contraceptive use on cerebrovascular reactivity. Q values are corrected for a false discovery rate (FDR) over 83 regions. **Supporting Table 5:** Sensitivity analysis of medication status on vascular outcomes.

## Data Availability

The data and code that support the findings of this study are openly available in g‐node at doi.org/10.12751/g-node.y6xp8z.
