# Supplementary material for: Cerebrovascular Function in Women With Polycystic Ovary Syndrome: A Pilot Multi‐Parameter Magnetic Resonance Imaging Study
Source: Clin Endocrinol (Oxf). 2025 Dec 1;104(5):493–506. doi: 10.1111/cen.70067 (PMC13040515; doi:10.1111/cen.70067)
Supplement: Supplementary file 1 — Supporting Figure 1: Violin plot comparing cerebral blood flow (CBF; ml/100g/min) values from all regions of interest (ROIs) between the healthy control group (left, green) and the PCOS group (right, blue). Supporting Figure 2: Violin plot comparing PETCO2 (defined as the max‐min end‐tidal CO2 trace recorded during the breath hold task) between the control group (left, green) and the PCOS group (right, blue). Supporting Table 1: Sensitivity analysis for outlier handling. Supporting Table 2: Regional interactions with PCOS status on cerebral blood flow. Q values are corrected for a false discovery rate (FDR) over 83 regions. Supporting Table 3: Regional interactions with PCOS status on cerebrovascular reactivity. Q values are corrected for a false discovery rate (FDR) over 83 regions. Supporting Table 4: Regional interactions with Hormonal contraceptive use on cerebrovascular reactivity. Q values are corrected for a false discovery rate (FDR) over 83 regions. Supporting Table 5: Sensitivity analysis of medication status on vascular outcomes. [file CEN-104-493-s001.docx]

**Supplemental material**

**Methods**

*MRI session – acquisition*

Data were acquired at Cardiff University Brain Research Imaging Centre (CUBRIC) on a Siemens MAGNETOM Prisma 3T scanner with a 32-channel head coil. A high-resolution structural T1 image was collected via an MPRAGE sequence (1mm^3^; repetition time [TR]=2.1s; echo time [TE]=3.24ms). The structure of carotid arteries was imaged using a limited FOV time of flight (TOF) scan (TR=40.65ms; TE=5.6ms; FA=10; 1 slab; voxel size=0.47*0.47*1.30mm). In order to assess CVR, an EPI scan was completed during a 9-minute breath hold task (TR=1s; TE=30ms; GRAPPA 2; Multiband 4; voxel size=2.0mm^3^). A gre fieldmap was also taken to aid in distortion correction.

Pulsatility Index (PI) in the internal carotid arteries was measured using dynamic inflow magnitude contrast ^DIMAC; 2^. A single-slice EPI was acquired, suppressing static signal and with the inflow signal in the flow-velocity regime ^2,3^. This provided flow-velocity-weighted images with a 15ms temporal resolution, allowing the pulse waveform to be resolved for individual heartbeats. The single EPI slice was acquired perpendicular to the internal carotid arteries (TR=15ms; TE=6.8ms; flip angle 90°; 2x2x10mm; 200x200 mm field of view; GRAPPA 5; phase partial Fourier 0.75; 4096 repetitions in 65s; slice thickness=10mm).

To estimate global oxygen extraction fraction (OEF) and cerebral metabolic rate of oxygen (CMRO_2_), a T2-relaxation-under-spin-tagging ^TRUST; 4,5^ sequence was used (TR=3s; TE=3.9ms). This was manually positioned over the sagittal sinus, using the AC-PC and occipital notch as navigational anatomical landmarks. A multi post labelling delay pseudocontinuous arterial spin labelling (MPLD-pCASL) perfusion scan was completed to assess cerebral blood flow (CBF) and arterial arrival time (AAT), with the tagging plane positioned perpendicular to the internal carotid arteries using the TOF scan as reference (maximum TR=5.6s; TE=11s; voxel resolution=3.4x3.4x6.0mm; tag duration=1500; post-labelling delays [PLDs]=250-3000ms in steps of 250ms; GRAPPA=2). To remove spatial signal inhomogeneity due to receive array coil sensitivity, Siemens in-built pre-scan normalizing correction was applied. To allow quantification, two separate equilibrium magnetization maps (M0 scan; phase encoding direction PA and AP) were also obtained, with TR=6000/1300ms and TE=11ms.

To monitor and measure physiological data during the scan, a pulse oximeter (Biopac Systems Inc, California, USA), respiratory belt and nasal cannula (AEI Technologies, PA, US) were secured. The head was secured with foam cushions to minimise movement and participants were instructed to stay as still as possible for the duration of the scan.

*MRI session – pre-processing*

*Global oxygen extraction fraction* *and cerebral metabolic rate of oxygen –* Both of these metrics were estimated using TRUST scan data. The T_2_ of blood was calculated by non-linear least squared fitting of a mono-exponential equation to the TRUST difference data, as a function of the effective echo times and T_2_. The two most intense voxels from a sagittal sinus ROI were used and venous oxygenation (Yv) was estimated by inverting the relationship between Yv, hemoglobin (Hb) and the T_2_ ^4^. OEF was calculated as:

$$OEF=(\frac{\left( SaO_{2}- SvO_{2} \right)}{SaO_{2}})$$

Global CMRO_2_ was then calculated as:

$$CMRO_{2}=gmCBF*CaO_{2}$$

Whereby gmCBF refers to the median CBF value (from the above perfusion maps) extracted using the individual’s grey matter mask, and CaO_2_ is defined as:

$$CaO_{2}=\left( \left[ Hb \right]*1.34* SaO_{2} \right)*OEF*39.34$$

SaO_2_ was assumed to be 98%, as has been used previously^12^.

**Results**

*Initial cerebral blood flow analysis*

Summary results for all ROIs are plotted by group in Figure S1. As in the main text, three iterations of a linear mixed model were used to examine the data.

*Status model.* This initial linear mixed model only had PCOS status and ROI as fixed effects, with participant ID as the random effect. PCOS status was not found to significantly contribute to the model (χ^2^(1)=1.39; p=0.24).

*Status/HC model.* Both PCOS status (χ^2^(1)=6.02; p=0.01) and HC (χ^2^(1)=6.57; p=0.01) use significantly contributed to the model. PCOS status was associated with a decrease of 10.56ml/100g/min±4.07 (SE), while HC use was associated with a decrease of 14.19ml/100g/min±5.20 (SE).

*Status/HC/testosterone/HOMA2-IR model.* PCOS status continued to have a significant contribution to the model (χ^2^(1)=5.86; p=0.02). PCOS status was associated with an estimated CBF decrease of 10.99ml/100g/min±4.29 (SE). HC was also statistically significant (χ^2^(1)=6.56; p=0.01) and was associated with a decrease of 14.17ml/100g/min±5.20 (SE). These factors therefore seemed to have a similar influence with or without the biochemical variables, suggesting other mechanisms. Neither testosterone (χ^2^(1)=0.08; p=0.78) nor HOMA2-IR (χ^2^(1)=0.01; p=0.98) significantly explained variance of the outcome measures.

However, upon closer inspection of the data and residual plots, an outlier in this ROI (label 74) was found (defined as 2 standard deviations from the mean for that ROI) which was implausibly high and likely having an undue effect on the results. This outlier was therefore removed and the analysis repeated, as reported in the main text.

We examined whether the pattern of results was robust to outlier exclusion. As a sensitivity analysis, CBF models were ran with a) the full sample, b) the sample excluding the outlier value, and c) the full sample with a robust mixed-effect model (Huber weighting). The pattern of the results (i.e., directionality of the estimates and standard errors) remained consistent, suggesting a degree of robustness to the results.

Figure S1 – Violin plot comparing cerebral blood flow (CBF; ml/100g/min) values from all regions of interest (ROIs) between the healthy control group (left, green) and the PCOS group (right, blue). The box-and-whisker plots indicate the median (central line), first and third quartile (hinges), and 1.5 * interquartile range from the hinge (whiskers). Single datapoints are outside this range.

*Table S1 – Sensitivity analysis for outlier handling*

|  |  | *Model 1* | *Model 2* | *Model 3* |
| --- | --- | --- | --- | --- |
|  | *Fixed effects* | *Estimate (SE)* | *Estimate (SE)* | *Estimate (SE)* |
| **Full sample** | *PCOS status* | -4.65 (3.89) | -7.37 (4.02) | -8.02 (4.32) |
|  | *Hormonal contraceptive use* |  | -10.87 (6.35) | -11.01 (6.37) |
|  | *Testosterone* |  |  | 1.79 (4.77) |
|  | *HOMA2-IR* |  |  | 0.09 (3.12) |
| **Sample without outlier** | *PCOS status* | -4.78 (3.88) | -7.50 (4.01) | -8.07 (4.31) |
|  | *Hormonal contraceptive use* |  | -10.87 (6.34) | -11.03 (6.37) |
|  | *Testosterone* |  |  | 1.50 (4.77) |
|  | *HOMA2-IR* |  |  | 0.25 (3.12) |
| **Robust model** | *PCOS status* | -3.38 (3.63) | -6.04 (3.85) | -6.68 (4.52) |
|  | *Hormonal contraceptive use* |  | -9.07 (6.24) | -9.18 (6.67) |
|  | *Testosterone* |  |  | 1.84 (5.00) |
|  | *HOMA2-IR* |  |  | 0.12 (3.27) |

*SE = Standard error.*

**Regional analysis**

83 regions were delineated via the Desikan-Killiany Atlas^24^ and, if an overall interaction with region-of-interest (ROI) was found to be statistically significant for a particular outcome, the individual coefficients were examined and a false discovery rate (FDR) correction for multiple comparisons was employed using the Benjamini-Hochberg (BH) procedure^28^ (83 per family). A statistically significant ROI interaction was found with PCOS status in terms of CBF value (*χ^2^*=110.31; p=0.029), as well as with PCOS status (*χ^2^*=154.25; p-value<0.001) and hormonal contraceptives (*χ^2^*=337.49; p-value<0.001). The full tables of individual ROI coefficients are presented in Tables S2-4.

*Table S2 – Regional interactions with PCOS status on cerebral blood flow. Q values are corrected for a false discovery rate (FDR) over 83 regions.*

| **Label** | **Estimate** | **Std. Error** | **df** | **t value** | **P value** | **Q value** |
| --- | --- | --- | --- | --- | --- | --- |
| lateralorbitofrontal_rh | -1.9672813 | 7.14098993 | 2227.00404 | -0.2754914 | 0.78296429 | 0.96086741 |
| parsorbitalis_rh | 4.09932307 | 7.14098993 | 2227.00404 | 0.57405529 | 0.56598836 | 0.96086741 |
| frontalpole_rh | -4.763592 | 7.14098993 | 2227.00404 | -0.6670773 | 0.50479189 | 0.96086741 |
| medialorbitofrontal_rh | 1.9828865 | 7.14098993 | 2227.00404 | 0.2776767 | 0.78128632 | 0.96086741 |
| parstriangularis_rh | 0.60277935 | 7.14098993 | 2227.00404 | 0.08441117 | 0.93273711 | 0.96771475 |
| parsopercularis_rh | -5.3018314 | 7.14098993 | 2227.00404 | -0.7424505 | 0.45789274 | 0.96086741 |
| rostralmiddlefrontal_rh | -3.3878466 | 7.14098993 | 2227.00404 | -0.4744225 | 0.63524513 | 0.96086741 |
| superiorfrontal_rh | 3.91620477 | 7.14098993 | 2227.00404 | 0.54841203 | 0.58346399 | 0.96086741 |
| caudalmiddlefrontal_rh | 2.06160887 | 7.14098993 | 2227.00404 | 0.28870071 | 0.77283729 | 0.96086741 |
| precentral_rh | 2.40342302 | 7.14098993 | 2227.00404 | 0.3365672 | 0.73647489 | 0.96086741 |
| paracentral_rh | 3.87185247 | 7.14098993 | 2227.00404 | 0.54220108 | 0.58773424 | 0.96086741 |
| rostralanteriorcingulate_rh | 2.45970228 | 7.14098993 | 2227.00404 | 0.34444836 | 0.73054165 | 0.96086741 |
| caudalanteriorcingulate_rh | -0.9129593 | 7.14098993 | 2227.00404 | -0.1278477 | 0.89828102 | 0.96086741 |
| posteriorcingulate_rh | -5.8094201 | 7.14098993 | 2227.00404 | -0.8135315 | 0.41600038 | 0.96086741 |
| isthmuscingulate_rh | -1.984024 | 7.14098993 | 2227.00404 | -0.277836 | 0.78116405 | 0.96086741 |
| postcentral_rh | 2.75670597 | 7.14098993 | 2227.00404 | 0.38603975 | 0.69950411 | 0.96086741 |
| supramarginal_rh | -1.5346925 | 7.14098993 | 2227.00404 | -0.2149131 | 0.82985473 | 0.96086741 |
| superiorparietal_rh | 0.7912396 | 7.14098993 | 2227.00404 | 0.11080251 | 0.91178294 | 0.96086741 |
| inferiorparietal_rh | -0.1989677 | 7.14098993 | 2227.00404 | -0.0278628 | 0.9777741 | 0.9777741 |
| precuneus_rh | -4.0677063 | 7.14098993 | 2227.00404 | -0.5696278 | 0.56898763 | 0.96086741 |
| cuneus_rh | 1.54950182 | 7.14098993 | 2227.00404 | 0.21698698 | 0.82823837 | 0.96086741 |
| pericalcarine_rh | 3.2646875 | 7.14098993 | 2227.00404 | 0.45717576 | 0.64758931 | 0.96086741 |
| lateraloccipital_rh | -0.7662292 | 7.14098993 | 2227.00404 | -0.1073001 | 0.91456054 | 0.96086741 |
| lingual_rh | -0.9751583 | 7.14098993 | 2227.00404 | -0.1365579 | 0.89139263 | 0.96086741 |
| fusiform_rh | 5.32732645 | 7.14098993 | 2227.00404 | 0.74602072 | 0.45573364 | 0.96086741 |
| parahippocampal_rh | 0.97719612 | 7.14098993 | 2227.00404 | 0.13684323 | 0.89116708 | 0.96086741 |
| entorhinal_rh | 3.54881023 | 7.14098993 | 2227.00404 | 0.49696334 | 0.61926396 | 0.96086741 |
| temporalpole_rh | 6.12911188 | 7.14098993 | 2227.00404 | 0.85830003 | 0.39081918 | 0.96086741 |
| inferiortemporal_rh | -1.3007844 | 7.14098993 | 2227.00404 | -0.1821574 | 0.85547575 | 0.96086741 |
| middletemporal_rh | -1.273406 | 7.14098993 | 2227.00404 | -0.1783235 | 0.85848518 | 0.96086741 |
| bankssts_rh | -3.8142046 | 7.14098993 | 2227.00404 | -0.5341283 | 0.59330611 | 0.96086741 |
| superiortemporal_rh | -1.6286835 | 7.14098993 | 2227.00404 | -0.2280753 | 0.81960863 | 0.96086741 |
| transversetemporal_rh | 1.10697303 | 7.14098993 | 2227.00404 | 0.15501675 | 0.87682216 | 0.96086741 |
| insula_rh | -1.5695972 | 7.14098993 | 2227.00404 | -0.2198011 | 0.82604623 | 0.96086741 |
| thalamusproper_rh | 1.28832282 | 7.14098993 | 2227.00404 | 0.18041236 | 0.85684527 | 0.96086741 |
| caudate_rh | -7.8082394 | 7.14098993 | 2227.00404 | -1.0934393 | 0.2743192 | 0.96086741 |
| putamen_rh | 1.18321863 | 7.14098993 | 2227.00404 | 0.16569392 | 0.86841287 | 0.96086741 |
| pallidum_rh | 4.40899308 | 7.14098993 | 2227.00404 | 0.61742043 | 0.53702057 | 0.96086741 |
| accumbensarea_rh | 3.42898042 | 7.14098993 | 2227.00404 | 0.48018278 | 0.63114459 | 0.96086741 |
| hippocampus_rh | 4.06503393 | 7.14098993 | 2227.00404 | 0.56925356 | 0.56924149 | 0.96086741 |
| amygdala_rh | -5.97294 | 7.14098993 | 2227.00404 | -0.8364302 | 0.40300264 | 0.96086741 |
| lateralorbitofrontal_lh | 4.29840283 | 7.14098993 | 2227.00404 | 0.60193375 | 0.54727951 | 0.96086741 |
| parsorbitalis_lh | -0.8916416 | 7.14098993 | 2227.00404 | -0.1248625 | 0.90064371 | 0.96086741 |
| frontalpole_lh | 0.28490857 | 7.14098993 | 2227.00404 | 0.03989763 | 0.96817832 | 0.9777741 |
| medialorbitofrontal_lh | -4.7695024 | 7.14098993 | 2227.00404 | -0.6679049 | 0.50426348 | 0.96086741 |
| parstriangularis_lh | -4.531297 | 7.14098993 | 2227.00404 | -0.6345475 | 0.52578879 | 0.96086741 |
| parsopercularis_lh | -1.7997226 | 7.14098993 | 2227.00404 | -0.252027 | 0.80104342 | 0.96086741 |
| rostralmiddlefrontal_lh | 2.6623405 | 7.14098993 | 2227.00404 | 0.37282513 | 0.70931408 | 0.96086741 |
| superiorfrontal_lh | 2.48598148 | 7.14098993 | 2227.00404 | 0.34812841 | 0.72777665 | 0.96086741 |
| caudalmiddlefrontal_lh | 8.55188205 | 7.14098993 | 2227.00404 | 1.19757655 | 0.23120936 | 0.96086741 |
| precentral_lh | -3.9915109 | 7.14098993 | 2227.00404 | -0.5589576 | 0.57624683 | 0.96086741 |
| paracentral_lh | 6.5767124 | 7.14098993 | 2227.00404 | 0.92098049 | 0.35716037 | 0.96086741 |
| rostralanteriorcingulate_lh | 0.32896125 | 7.14098993 | 2227.00404 | 0.04606662 | 0.96326128 | 0.9777741 |
| caudalanteriorcingulate_lh | -8.2661022 | 7.14098993 | 2227.00404 | -1.1575569 | 0.24716904 | 0.96086741 |
| posteriorcingulate_lh | -4.7562308 | 7.14098993 | 2227.00404 | -0.6660464 | 0.50545041 | 0.96086741 |
| isthmuscingulate_lh | -9.2633066 | 7.14098993 | 2227.00404 | -1.297202 | 0.19469605 | 0.96086741 |
| postcentral_lh | -0.848857 | 7.14098993 | 2227.00404 | -0.1188711 | 0.90538824 | 0.96086741 |
| supramarginal_lh | -8.458056 | 7.14098993 | 2227.00404 | -1.1844375 | 0.23636633 | 0.96086741 |
| superiorparietal_lh | -2.5472263 | 7.14098993 | 2227.00404 | -0.3567049 | 0.72134651 | 0.96086741 |
| inferiorparietal_lh | 3.0799017 | 7.14098993 | 2227.00404 | 0.43129898 | 0.66629273 | 0.96086741 |
| precuneus_lh | -2.2525143 | 7.14098993 | 2227.00404 | -0.3154345 | 0.75246136 | 0.96086741 |
| cuneus_lh | 3.72454508 | 7.14098993 | 2227.00404 | 0.52157266 | 0.60201977 | 0.96086741 |
| pericalcarine_lh | -17.706915 | 7.14098993 | 2227.00404 | -2.4796163 | 0.01322585 | 0.54887265 |
| lateraloccipital_lh | -3.6736245 | 7.14098993 | 2227.00404 | -0.5144419 | 0.60699411 | 0.96086741 |
| lingual_lh | 3.42894752 | 7.14098993 | 2227.00404 | 0.48017818 | 0.63114786 | 0.96086741 |
| fusiform_lh | -1.1459588 | 7.14098993 | 2227.00404 | -0.1604762 | 0.87252054 | 0.96086741 |
| parahippocampal_lh | 2.03601463 | 7.42720835 | 2227.08048 | 0.27412919 | 0.78401076 | 0.96086741 |
| entorhinal_lh | 3.17020229 | 7.39453543 | 2227.07662 | 0.42872231 | 0.66816679 | 0.96086741 |
| temporalpole_lh | 15.40264 | 7.42721182 | 2227.07885 | 2.07381187 | 0.03821147 | 0.96086741 |
| inferiortemporal_lh | 5.44848843 | 7.14098993 | 2227.00404 | 0.76298783 | 0.44555146 | 0.96086741 |
| middletemporal_lh | 5.06252595 | 7.14098993 | 2227.00404 | 0.70893896 | 0.47843656 | 0.96086741 |
| bankssts_lh | 3.26887582 | 7.14098993 | 2227.00404 | 0.45776228 | 0.6471679 | 0.96086741 |
| superiortemporal_lh | 7.5414391 | 7.14098993 | 2227.00404 | 1.05607754 | 0.29104734 | 0.96086741 |
| transversetemporal_lh | -29.04983 | 7.2634508 | 2227.0403 | -3.999453 | 6.56E-05 | 0.00544143 |
| insula_lh | -3.8173251 | 7.14098993 | 2227.00404 | -0.5345653 | 0.59300387 | 0.96086741 |
| thalamusproper_lh | -1.6508703 | 7.14098993 | 2227.00404 | -0.2311823 | 0.81719444 | 0.96086741 |
| caudate_lh | -4.1533621 | 7.14098993 | 2227.00404 | -0.5816227 | 0.56087969 | 0.96086741 |
| putamen_lh | -1.4606641 | 7.14098993 | 2227.00404 | -0.2045464 | 0.8379452 | 0.96086741 |
| pallidum_lh | 9.69022123 | 7.14098993 | 2227.00404 | 1.3569857 | 0.17492327 | 0.96086741 |
| accumbensarea_lh | -12.296563 | 7.14098993 | 2227.00404 | -1.7219689 | 0.085214 | 0.96086741 |
| hippocampus_lh | 9.06861582 | 7.14098993 | 2227.00404 | 1.26993819 | 0.20423932 | 0.96086741 |
| amygdala_lh | -4.641719 | 7.19811974 | 2227.02368 | -0.6448516 | 0.51908978 | 0.96086741 |
| brainstem_lh | 1.78528715 | 7.14098993 | 2227.00404 | 0.25000555 | 0.80260611 | 0.96086741 |

*Table S3 – Regional interactions with PCOS status on cerebrovascular reactivity. Q values are corrected for a false discovery rate (FDR) over 83 regions.*

| **Label** | **Estimate** | **Std. Error** | **df** | **t value** | **P value** | **Q value** |
| --- | --- | --- | --- | --- | --- | --- |
| lateralorbitofrontal_rh | -0.5051443 | 0.24274292 | 1966.01732 | -2.0809847 | 0.03756446 | 0.51964175 |
| parsorbitalis_rh | -0.1538966 | 0.23869993 | 1965.91442 | -0.6447282 | 0.51917859 | 0.98991295 |
| frontalpole_rh | -0.1014248 | 0.23869993 | 1965.91442 | -0.4249049 | 0.67095251 | 0.98991295 |
| medialorbitofrontal_rh | 0.1354863 | 0.23869993 | 1965.91442 | 0.56760092 | 0.5703708 | 0.98991295 |
| parstriangularis_rh | 0.029999 | 0.23869993 | 1965.91442 | 0.12567662 | 0.90000076 | 0.98991295 |
| parsopercularis_rh | 0.22535337 | 0.23869993 | 1965.91442 | 0.94408643 | 0.34524149 | 0.98991295 |
| rostralmiddlefrontal_rh | -0.0235556 | 0.23869993 | 1965.91442 | -0.0986828 | 0.92140022 | 0.98991295 |
| superiorfrontal_rh | -0.0231575 | 0.23869993 | 1965.91442 | -0.097015 | 0.9227244 | 0.98991295 |
| caudalmiddlefrontal_rh | 0.01758203 | 0.23869993 | 1965.91442 | 0.07365747 | 0.94129043 | 0.98991295 |
| precentral_rh | -0.0575016 | 0.23869993 | 1965.91442 | -0.2408948 | 0.80966179 | 0.98991295 |
| paracentral_rh | -0.0427365 | 0.23869993 | 1965.91442 | -0.1790385 | 0.85792596 | 0.98991295 |
| rostralanteriorcingulate_rh | -0.1692862 | 0.23869993 | 1965.91442 | -0.7092009 | 0.47828393 | 0.98991295 |
| caudalanteriorcingulate_rh | -0.0229342 | 0.23869993 | 1965.91442 | -0.0960798 | 0.92346701 | 0.98991295 |
| posteriorcingulate_rh | -0.0702133 | 0.23869993 | 1965.91442 | -0.2941489 | 0.76867514 | 0.98991295 |
| isthmuscingulate_rh | -0.1221107 | 0.23869993 | 1965.91442 | -0.5115656 | 0.60901254 | 0.98991295 |
| postcentral_rh | -0.1657861 | 0.24274292 | 1966.01732 | -0.6829697 | 0.49470645 | 0.98991295 |
| supramarginal_rh | 0.00762123 | 0.23869993 | 1965.91442 | 0.03192809 | 0.97453264 | 0.98991295 |
| superiorparietal_rh | 0.0294288 | 0.23869993 | 1965.91442 | 0.12328784 | 0.90189177 | 0.98991295 |
| inferiorparietal_rh | -0.0521542 | 0.24274292 | 1966.01732 | -0.2148536 | 0.82990373 | 0.98991295 |
| precuneus_rh | -0.1217907 | 0.23869993 | 1965.91442 | -0.510225 | 0.60995114 | 0.98991295 |
| cuneus_rh | -0.1456148 | 0.24274292 | 1966.01732 | -0.5998726 | 0.5486603 | 0.98991295 |
| pericalcarine_rh | -0.2058149 | 0.23869993 | 1965.91442 | -0.8622326 | 0.38866469 | 0.98991295 |
| lateraloccipital_rh | -0.121627 | 0.24274292 | 1966.01732 | -0.5010527 | 0.61639013 | 0.98991295 |
| lingual_rh | -0.3942883 | 0.23869993 | 1965.91442 | -1.6518159 | 0.09873179 | 0.98991295 |
| fusiform_rh | -0.3760772 | 0.24274292 | 1966.01732 | -1.5492818 | 0.12147493 | 0.98991295 |
| parahippocampal_rh | 0.0201421 | 0.24274292 | 1966.01732 | 0.08297707 | 0.93387822 | 0.98991295 |
| entorhinal_rh | 0.01963178 | 0.24274292 | 1966.01732 | 0.0808748 | 0.93554976 | 0.98991295 |
| temporalpole_rh | 0.00301817 | 0.23869993 | 1965.91442 | 0.01264419 | 0.98991295 | 0.98991295 |
| inferiortemporal_rh | -0.115485 | 0.24274292 | 1966.01732 | -0.4757502 | 0.6343052 | 0.98991295 |
| middletemporal_rh | -0.1491487 | 0.24274292 | 1966.01732 | -0.6144309 | 0.53900172 | 0.98991295 |
| bankssts_rh | -0.1006217 | 0.24274292 | 1966.01732 | -0.4145198 | 0.67853868 | 0.98991295 |
| superiortemporal_rh | 0.11225167 | 0.23869993 | 1965.91442 | 0.47026267 | 0.63821953 | 0.98991295 |
| transversetemporal_rh | -0.125896 | 0.23869993 | 1965.91442 | -0.5274236 | 0.597959 | 0.98991295 |
| insula_rh | -0.0659452 | 0.24274292 | 1966.01732 | -0.271667 | 0.78590661 | 0.98991295 |
| thalamusproper_rh | 0.11713653 | 0.23869993 | 1965.91442 | 0.49072713 | 0.62367422 | 0.98991295 |
| caudate_rh | -0.0319345 | 0.24274292 | 1966.01732 | -0.1315569 | 0.89534824 | 0.98991295 |
| putamen_rh | 0.1258097 | 0.23869993 | 1965.91442 | 0.52706215 | 0.5982099 | 0.98991295 |
| pallidum_rh | 0.1220637 | 0.23869993 | 1965.91442 | 0.51136881 | 0.60915026 | 0.98991295 |
| accumbensarea_rh | -0.0728564 | 0.24274292 | 1966.01732 | -0.3001383 | 0.76410342 | 0.98991295 |
| hippocampus_rh | 0.04550867 | 0.23869993 | 1965.91442 | 0.1906522 | 0.84881778 | 0.98991295 |
| amygdala_rh | 0.00560923 | 0.23869993 | 1965.91442 | 0.0234991 | 0.98125454 | 0.98991295 |
| lateralorbitofrontal_lh | -0.0828313 | 0.24274292 | 1966.01732 | -0.3412307 | 0.7329664 | 0.98991295 |
| parsorbitalis_lh | -0.0625387 | 0.23869993 | 1965.91442 | -0.2619973 | 0.79335099 | 0.98991295 |
| frontalpole_lh | 0.1384857 | 0.23869993 | 1965.91442 | 0.58016648 | 0.56186884 | 0.98991295 |
| medialorbitofrontal_lh | -0.0839497 | 0.24274292 | 1966.01732 | -0.3458381 | 0.72950139 | 0.98991295 |
| parstriangularis_lh | -0.1674892 | 0.24274292 | 1966.01732 | -0.6899858 | 0.49028454 | 0.98991295 |
| parsopercularis_lh | -0.1761772 | 0.24274292 | 1966.01732 | -0.725777 | 0.46806185 | 0.98991295 |
| rostralmiddlefrontal_lh | 0.07738953 | 0.23869993 | 1965.91442 | 0.32421263 | 0.74581155 | 0.98991295 |
| superiorfrontal_lh | -0.0184177 | 0.24274292 | 1966.01732 | -0.0758732 | 0.9395277 | 0.98991295 |
| caudalmiddlefrontal_lh | -0.0479869 | 0.23869993 | 1965.91442 | -0.2010344 | 0.84069246 | 0.98991295 |
| precentral_lh | -0.0637341 | 0.23869993 | 1965.91442 | -0.267005 | 0.78949334 | 0.98991295 |
| paracentral_lh | -0.1050491 | 0.24764883 | 1966.00167 | -0.4241856 | 0.67147688 | 0.98991295 |
| rostralanteriorcingulate_lh | 0.01600364 | 0.24443472 | 1966.02979 | 0.06547205 | 0.94780481 | 0.98991295 |
| caudalanteriorcingulate_lh | -0.3302432 | 0.2476448 | 1966.0684 | -1.3335359 | 0.18251047 | 0.98991295 |
| posteriorcingulate_lh | 0.11655807 | 0.24443393 | 1966.03042 | 0.47684897 | 0.63352267 | 0.98991295 |
| isthmuscingulate_lh | -0.0692933 | 0.24274292 | 1966.01732 | -0.2854595 | 0.77532224 | 0.98991295 |
| postcentral_lh | -0.1685726 | 0.24274292 | 1966.01732 | -0.6944493 | 0.48748257 | 0.98991295 |
| supramarginal_lh | -0.0804664 | 0.24042015 | 1965.92969 | -0.3346907 | 0.73789413 | 0.98991295 |
| superiorparietal_lh | -0.0059709 | 0.23869993 | 1965.91442 | -0.0250143 | 0.98004614 | 0.98991295 |
| inferiorparietal_lh | -0.0705135 | 0.24443463 | 1966.03227 | -0.2884759 | 0.7730129 | 0.98991295 |
| precuneus_lh | -0.0384076 | 0.24274292 | 1966.01732 | -0.1582232 | 0.87429714 | 0.98991295 |
| cuneus_lh | -0.0629084 | 0.24764883 | 1966.00167 | -0.2540224 | 0.79950479 | 0.98991295 |
| pericalcarine_lh | 1.03625003 | 0.25542917 | 1966.07593 | 4.05689781 | 5.17E-05 | 0.0021444 |
| lateraloccipital_lh | -0.1020272 | 0.24274292 | 1966.01732 | -0.4203096 | 0.67430525 | 0.98991295 |
| lingual_lh | -0.0908234 | 0.24274292 | 1966.01732 | -0.3741547 | 0.70832953 | 0.98991295 |
| fusiform_lh | -0.1055851 | 0.24274292 | 1966.01732 | -0.4349668 | 0.66363428 | 0.98991295 |
| parahippocampal_lh | 1.37062325 | 0.26852563 | 1966.20402 | 5.10425487 | 3.64E-07 | 3.02E-05 |
| entorhinal_lh | 0.64958007 | 0.28017767 | 1966.20867 | 2.31845764 | 0.02052657 | 0.34074109 |
| temporalpole_lh | -0.4926335 | 0.25728359 | 1966.06599 | -1.9147488 | 0.05566952 | 0.66008149 |
| inferiortemporal_lh | -0.0880209 | 0.24274292 | 1966.01732 | -0.3626097 | 0.71693547 | 0.98991295 |
| middletemporal_lh | -0.3337319 | 0.24274292 | 1966.01732 | -1.3748366 | 0.16933876 | 0.98991295 |
| bankssts_lh | 0.10500253 | 0.25943134 | 1966.10294 | 0.40474113 | 0.68571184 | 0.98991295 |
| superiortemporal_lh | -0.0746558 | 0.24274292 | 1966.01732 | -0.3075509 | 0.75845669 | 0.98991295 |
| transversetemporal_lh | -0.0793893 | 0.25538876 | 1966.06583 | -0.3108565 | 0.75594263 | 0.98991295 |
| insula_lh | -0.0208644 | 0.23869993 | 1965.91442 | -0.0874083 | 0.93035584 | 0.98991295 |
| thalamusproper_lh | 0.14877471 | 0.24438853 | 1965.96504 | 0.60876305 | 0.54275184 | 0.98991295 |
| caudate_lh | -0.0886611 | 0.24274292 | 1966.01732 | -0.3652467 | 0.71496652 | 0.98991295 |
| putamen_lh | 0.06623253 | 0.25120092 | 1966.07051 | 0.26366356 | 0.79206681 | 0.98991295 |
| pallidum_lh | 0.33658624 | 0.3278092 | 1966.43581 | 1.02677483 | 0.30465279 | 0.98991295 |
| accumbensarea_lh | 0.59399862 | 0.2559747 | 1966.13371 | 2.32053646 | 0.02041374 | 0.34074109 |
| hippocampus_lh | -0.6817569 | 0.24443484 | 1966.03438 | -2.7891151 | 0.00533616 | 0.14763369 |
| amygdala_lh | -0.0071475 | 0.24764883 | 1966.00167 | -0.0288613 | 0.97697817 | 0.98991295 |
| brainstem_lh | 0.17470597 | 0.23869993 | 1965.91442 | 0.73190623 | 0.46431304 | 0.98991295 |

*Table S4 – Regional interactions with Hormonal contraceptive use on cerebrovascular reactivity. Q values are corrected for a false discovery rate (FDR) over 83 regions.*

| **Label** | **Estimate** | **Std. Error** | **df** | **t value** | **P value** | **Q value** |
| --- | --- | --- | --- | --- | --- | --- |
| lateralorbitofrontal_rh | 0.24098659 | 0.39824127 | 1966.64974 | 0.6051271 | 0.54516436 | 0.8701662 |
| parsorbitalis_rh | 0.23644762 | 0.35975063 | 1965.88207 | 0.65725423 | 0.5110944 | 0.86516296 |
| frontalpole_rh | -0.00328 | 0.35975063 | 1965.88207 | -0.0091173 | 0.99272644 | 0.993903 |
| medialorbitofrontal_rh | -0.4121031 | 0.35975063 | 1965.88207 | -1.1455244 | 0.25213142 | 0.74532594 |
| parstriangularis_rh | -0.2838603 | 0.35975063 | 1965.88207 | -0.7890474 | 0.43017944 | 0.86516296 |
| parsopercularis_rh | -0.1811022 | 0.35975063 | 1965.88207 | -0.5034103 | 0.61473221 | 0.87617577 |
| rostralmiddlefrontal_rh | -0.1832685 | 0.35975063 | 1965.88207 | -0.5094321 | 0.61050656 | 0.87617577 |
| superiorfrontal_rh | -0.2935004 | 0.35975063 | 1965.88207 | -0.8158441 | 0.41468812 | 0.86516296 |
| caudalmiddlefrontal_rh | 0.18790714 | 0.35975063 | 1965.88207 | 0.52232608 | 0.60150216 | 0.87617577 |
| precentral_rh | 0.1412583 | 0.35975063 | 1965.88207 | 0.39265617 | 0.69461602 | 0.90851402 |
| paracentral_rh | 0.38786062 | 0.35975063 | 1965.88207 | 1.07813744 | 0.2811047 | 0.75263515 |
| rostralanteriorcingulate_rh | 0.32833817 | 0.35975063 | 1965.88207 | 0.91268268 | 0.36152137 | 0.83350759 |
| caudalanteriorcingulate_rh | 0.06914868 | 0.35975063 | 1965.88207 | 0.19221282 | 0.84759536 | 0.95905982 |
| posteriorcingulate_rh | 0.22508402 | 0.35975063 | 1965.88207 | 0.62566678 | 0.53160616 | 0.86516296 |
| isthmuscingulate_rh | 0.59193038 | 0.35975063 | 1965.88207 | 1.64539082 | 0.1000491 | 0.7288528 |
| postcentral_rh | 0.50764154 | 0.39824127 | 1966.64974 | 1.27470852 | 0.20256301 | 0.74532594 |
| supramarginal_rh | 0.19941291 | 0.35975063 | 1965.88207 | 0.55430872 | 0.57943063 | 0.87617577 |
| superiorparietal_rh | 0.26701226 | 0.35975063 | 1965.88207 | 0.74221485 | 0.45804584 | 0.86516296 |
| inferiorparietal_rh | 0.553996 | 0.39824127 | 1966.64974 | 1.39110644 | 0.16435051 | 0.74532594 |
| precuneus_rh | 0.64226205 | 0.35975063 | 1965.88207 | 1.78529792 | 0.07436713 | 0.7288528 |
| cuneus_rh | 0.393952 | 0.39824127 | 1966.64974 | 0.98922945 | 0.32267258 | 0.81991948 |
| pericalcarine_rh | -0.0060687 | 0.35975063 | 1965.88207 | -0.0168691 | 0.9865428 | 0.993903 |
| lateraloccipital_rh | 0.79089672 | 0.39824127 | 1966.64974 | 1.98597378 | 0.04717487 | 0.72629081 |
| lingual_rh | 0.88780083 | 0.35975063 | 1965.88207 | 2.46782289 | 0.01367835 | 0.47635492 |
| fusiform_rh | 0.38568659 | 0.39824127 | 1966.64974 | 0.96847468 | 0.33292645 | 0.81991948 |
| parahippocampal_rh | -0.1824669 | 0.39824127 | 1966.64974 | -0.4581817 | 0.64687252 | 0.8801708 |
| entorhinal_rh | -0.480115 | 0.39824127 | 1966.64974 | -1.2055884 | 0.22812127 | 0.74532594 |
| temporalpole_rh | -0.2469184 | 0.35975063 | 1965.88207 | -0.6863599 | 0.49256712 | 0.86516296 |
| inferiortemporal_rh | -0.1959068 | 0.39824127 | 1966.64974 | -0.49193 | 0.62282374 | 0.87617577 |
| middletemporal_rh | 0.49009713 | 0.39824127 | 1966.64974 | 1.23065379 | 0.21859954 | 0.74532594 |
| bankssts_rh | -0.0052985 | 0.39824127 | 1966.64974 | -0.0133049 | 0.98938591 | 0.993903 |
| superiortemporal_rh | -0.2018705 | 0.35975063 | 1965.88207 | -0.56114 | 0.57476608 | 0.87617577 |
| transversetemporal_rh | 0.22901986 | 0.35975063 | 1965.88207 | 0.63660727 | 0.52445481 | 0.86516296 |
| insula_rh | -0.1639971 | 0.39824127 | 1966.64974 | -0.4118035 | 0.68052835 | 0.90851402 |
| thalamusproper_rh | -0.3411989 | 0.35975063 | 1965.88207 | -0.9484318 | 0.34302626 | 0.81991948 |
| caudate_rh | -0.3278325 | 0.39824127 | 1966.64974 | -0.8232006 | 0.41049373 | 0.86516296 |
| putamen_rh | -0.577811 | 0.35975063 | 1965.88207 | -1.6061433 | 0.10840304 | 0.7288528 |
| pallidum_rh | 0.05966971 | 0.35975063 | 1965.88207 | 0.16586409 | 0.86828096 | 0.95905982 |
| accumbensarea_rh | -0.0502531 | 0.39824127 | 1966.64974 | -0.1261877 | 0.89959626 | 0.95905982 |
| hippocampus_rh | 0.00274939 | 0.35975063 | 1965.88207 | 0.0076425 | 0.993903 | 0.993903 |
| amygdala_rh | 0.23171064 | 0.35975063 | 1965.88207 | 0.64408682 | 0.51959428 | 0.86516296 |
| lateralorbitofrontal_lh | -0.0570387 | 0.39824127 | 1966.64974 | -0.1432265 | 0.88612601 | 0.95905982 |
| parsorbitalis_lh | 0.11604217 | 0.35975063 | 1965.88207 | 0.32256279 | 0.74706068 | 0.95393902 |
| frontalpole_lh | -0.8688398 | 0.35975063 | 1965.88207 | -2.4151167 | 0.01582089 | 0.47635492 |
| medialorbitofrontal_lh | -0.0978427 | 0.39824127 | 1966.64974 | -0.2456871 | 0.80595012 | 0.95905982 |
| parstriangularis_lh | 0.55624045 | 0.39824127 | 1966.64974 | 1.39674235 | 0.16264866 | 0.74532594 |
| parsopercularis_lh | 0.34779831 | 0.39824127 | 1966.64974 | 0.87333569 | 0.38258676 | 0.85823516 |
| rostralmiddlefrontal_lh | -0.4146702 | 0.35975063 | 1965.88207 | -1.1526601 | 0.24919004 | 0.74532594 |
| superiorfrontal_lh | -0.0595444 | 0.39824127 | 1966.64974 | -0.1495184 | 0.88115989 | 0.95905982 |
| caudalmiddlefrontal_lh | 0.06833194 | 0.35975063 | 1965.88207 | 0.18994252 | 0.84937379 | 0.95905982 |
| precentral_lh | 0.07175929 | 0.35975063 | 1965.88207 | 0.19946953 | 0.84191611 | 0.95905982 |
| paracentral_lh | -0.047541 | 0.49508345 | 1966.80185 | -0.0960262 | 0.92350951 | 0.97026948 |
| rostralanteriorcingulate_lh | -0.2127462 | 0.39871113 | 1966.6496 | -0.5335849 | 0.59368905 | 0.87617577 |
| caudalanteriorcingulate_lh | 0.18270476 | 0.39871115 | 1966.65058 | 0.45823841 | 0.64683179 | 0.8801708 |
| posteriorcingulate_lh | -0.0494617 | 0.39871087 | 1966.65043 | -0.1240541 | 0.90128513 | 0.95905982 |
| isthmuscingulate_lh | 0.28610009 | 0.39824127 | 1966.64974 | 0.71840893 | 0.47259052 | 0.86516296 |
| postcentral_lh | -0.0804614 | 0.39824127 | 1966.64974 | -0.2020418 | 0.83990497 | 0.95905982 |
| supramarginal_lh | 0.28885979 | 0.36027064 | 1965.8851 | 0.80178555 | 0.42277392 | 0.86516296 |
| superiorparietal_lh | 0.44051505 | 0.35975063 | 1965.88207 | 1.22450112 | 0.22090996 | 0.74532594 |
| inferiorparietal_lh | 0.61529756 | 0.39871099 | 1966.65219 | 1.54321696 | 0.12293903 | 0.7288528 |
| precuneus_lh | 0.64223918 | 0.39824127 | 1966.64974 | 1.61268865 | 0.10697261 | 0.7288528 |
| cuneus_lh | 0.32348904 | 0.49508345 | 1966.80185 | 0.65340306 | 0.51357285 | 0.86516296 |
| pericalcarine_lh | 0.19073728 | 0.49587683 | 1966.80123 | 0.38464649 | 0.70054093 | 0.90851402 |
| lateraloccipital_lh | 0.68272709 | 0.39824127 | 1966.64974 | 1.71435543 | 0.08662103 | 0.7288528 |
| lingual_lh | -0.265411 | 0.39824127 | 1966.64974 | -0.6664579 | 0.50519662 | 0.86516296 |
| fusiform_lh | 0.44596186 | 0.39824127 | 1966.64974 | 1.11982833 | 0.26292366 | 0.74532594 |
| parahippocampal_lh | -0.4547846 | 0.40384094 | 1966.65654 | -1.1261479 | 0.26024034 | 0.74532594 |
| entorhinal_lh | -1.1446289 | 0.49877374 | 1966.80539 | -2.2948861 | 0.02184451 | 0.47635492 |
| temporalpole_lh | 0.88893054 | 0.49633558 | 1966.80163 | 1.79098694 | 0.07344911 | 0.7288528 |
| inferiortemporal_lh | 0.77265204 | 0.39824127 | 1966.64974 | 1.94016064 | 0.05250295 | 0.72629081 |
| middletemporal_lh | 0.57028113 | 0.39824127 | 1966.64974 | 1.43199907 | 0.15230299 | 0.74532594 |
| bankssts_lh | -0.0887504 | 0.49684491 | 1966.80183 | -0.178628 | 0.85824823 | 0.95905982 |
| superiortemporal_lh | -0.3755785 | 0.39824127 | 1966.64974 | -0.943093 | 0.34574918 | 0.81991948 |
| transversetemporal_lh | -0.1207536 | 0.39948856 | 1966.23838 | -0.3022704 | 0.76247794 | 0.95887378 |
| insula_lh | -0.4007714 | 0.35975063 | 1965.88207 | -1.1140255 | 0.26540442 | 0.74532594 |
| thalamusproper_lh | 0.42668218 | 0.36084194 | 1965.88824 | 1.18246284 | 0.2371651 | 0.74532594 |
| caudate_lh | 0.0741205 | 0.39824127 | 1966.64974 | 0.18611957 | 0.85237019 | 0.95905982 |
| putamen_lh | -0.5550851 | 0.39979674 | 1966.64794 | -1.3884183 | 0.16516694 | 0.74532594 |
| pallidum_lh | -0.630205 | 0.50451819 | 1966.8035 | -1.2491225 | 0.21176892 | 0.74532594 |
| accumbensarea_lh | -0.9129549 | 0.40113358 | 1966.66065 | -2.2759373 | 0.02295686 | 0.47635492 |
| hippocampus_lh | 0.44048336 | 0.39871089 | 1966.65547 | 1.10476882 | 0.26939492 | 0.74532594 |
| amygdala_lh | -0.7727143 | 0.49508345 | 1966.80185 | -1.5607758 | 0.11873754 | 0.7288528 |
| brainstem_lh | 0.26622415 | 0.35975063 | 1965.88207 | 0.74002415 | 0.45937371 | 0.86516296 |

**Sensitivity analysis**

Sensitivity analysis was carried out for medicated compared to non-medicated individuals. For each vascular outcome, linear models were constructed in which medication status was a primary fixed effect, participant was a random effect and, if relevant, ROI/laterality was added as an additional fixed effect. We then examined whether medication status explained a significant amount of outcome variance. Results for this analysis are shown in table S5.

*Table S5 – Sensitivity analysis of medication status on vascular outcomes.*

| Outcomes | t-statistic | p-value | Estimate (SE) |
| --- | --- | --- | --- |
| OEF | 0.12 | 0.91 | 0.004 (0.03) |
| CMRO_2_ | -1.32 | 0.20 | -14.71 (11.11) |
| CBF | -0.688 | 0.50 | -2.80 (4.07) |
| AAT | -0.02 | 0.99 | -2.99 (2.00) |
| PI | 1.97 | 0.06 | 0.08 (0.04) |
| CVR | -1.694 | 0.10 | -3.41 (2.01) |

*OEF= oxygen extraction fraction, CMRO_2_= cerebral metabolic rate of oxygen, CBF= cerebral blood flow, AAT= arterial arrival time, PI= pulsatility index, CVR= cerebrovascular reactivity, SE= Standard Error, Df= Degrees-of-freedom.*

**
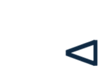
**

Figure S2 – Violin plot comparing PETCO_2_ (defined as the max-min end-tidal CO2 trace recorded during the breath hold task) between the control group (left, green) and the PCOS group (right, blue). The box-and-whisker plots indicate the median (central line), first and third quartile (hinges), and 1.5 * interquartile range from the hinge (whiskers). Individual datapoints are shown as filled circles. Data presented are from 15 PCOS patients and 10 healthy controls,

**Supplemental references**

1. Bull FC, Al-Ansari SS, Biddle S, et al. World Health Organization 2020 guidelines on physical activity and sedentary behaviour. *Br J Sports Med*. 2020;54(24):1451-1462. doi:10.1136/bjsports-2020-102955

2. Whittaker JR, Fasano F, Venzi M, et al. Measuring Arterial Pulsatility With Dynamic Inflow Magnitude Contrast. *Front Neurosci*. 2022;15:795749. doi:10.3389/fnins.2021.795749

3. Bianciardi M, Toschi N, Polimeni JR, et al. The pulsatility volume index: an indicator of cerebrovascular compliance based on fast magnetic resonance imaging of cardiac and respiratory pulsatility. *Philos Trans R Soc Math Phys Eng Sci*. 2016;374(2067):20150184. doi:10.1098/rsta.2015.0184

4. Lu H, Ge Y. Quantitative evaluation of oxygenation in venous vessels using T2‐Relaxation‐Under‐Spin‐Tagging MRI. *Magn Reson Med*. 2008;60(2):357-363. doi:10.1002/mrm.21627

5. Lu H, Xu F, Grgac K, Liu P, Qin Q, Van Zijl P. Calibration and validation of TRUST MRI for the estimation of cerebral blood oxygenation. *Magn Reson Med*. 2012;67(1):42-49. doi:10.1002/mrm.22970

6. Cox RW. AFNI: Software for Analysis and Visualization of Functional Magnetic Resonance Neuroimages. *Comput Biomed Res*. 1996;29(3):162-173. doi:10.1006/cbmr.1996.0014

7. Pinto J, Chappell MA, Okell TW, et al. Calibration of arterial spin labeling data—potential pitfalls in post‐processing. *Magn Reson Med*. 2020;83(4):1222-1234. doi:10.1002/mrm.28000

8. Herscovitch P, Raichle ME. What is the Correct Value for the Brain-Blood Partition Coefficient for Water? *J Cereb Blood Flow Metab*. 1985;5(1):65-69. doi:10.1038/jcbfm.1985.9

9. Zhao JM, Clingman CS, Närväinen MJ, Kauppinen RA, Van Zijl PCM. Oxygenation and hematocrit dependence of transverse relaxation rates of blood at 3T. *Magn Reson Med*. 2007;58(3):592-597. doi:10.1002/mrm.21342

10. Jenkinson M, Beckmann CF, Behrens TEJ, Woolrich MW, Smith SM. FSL. *NeuroImage*. 2012;62(2):782-790.

11. Desikan RS, Ségonne F, Fischl B, et al. An automated labeling system for subdividing the human cerebral cortex on MRI scans into gyral based regions of interest. *NeuroImage*. 2006;31(3):968-980. doi:10.1016/j.neuroimage.2006.01.021

12. Chandler HL, Stickland RC, Patitucci E, et al. Reduced brain oxygen metabolism in patients with multiple sclerosis: Evidence from dual-calibrated functional MRI. *J Cereb Blood Flow Metab*. 2023;43(1):115-128. doi:10.1177/0271678X221121849
